# Identification of new GLUT2-selective inhibitors through in silico ligand screening and validation in eukaryotic expression systems

**DOI:** 10.1038/s41598-021-93063-5

**Published:** 2021-07-02

**Authors:** Sina Schmidl, Oleg Ursu, Cristina V. Iancu, Mislav Oreb, Tudor I. Oprea, Jun-yong Choe

**Affiliations:** 1 Institute of Molecular Biosciences, Faculty of Biological Sciences, Goethe University Frankfurt, Frankfurt am Main, Germany; 2 Translational Informatics Division, Department of Internal Medicine, The University of New Mexico School of Medicine, Albuquerque, NM 87131 USA; 3 Department of Chemistry, East Carolina Diabetes and Obesity Institute, East Carolina University, Greenville, NC 27834 USA; 4 UNM Comprehensive Cancer Center, The University of New Mexico, Albuquerque, NM 87131 USA; 5 Department of Biochemistry and Molecular Biology, The Chicago Medical School, Rosalind Franklin University of Medicine and Science, North Chicago, IL 60064 USA; 6 Present Address: Computational and Structural Chemistry, Merck & Co., Inc., 2000 Galloping Hill Road, Kenilworth, NJ 07033 USA

**Keywords:** Membrane proteins, Virtual screening, Drug discovery and development

## Abstract

Glucose is an essential energy source for cells. In humans, its passive diffusion through the cell membrane is facilitated by members of the glucose transporter family (GLUT, SLC2 gene family). GLUT2 transports both glucose and fructose with low affinity and plays a critical role in glucose sensing mechanisms. Alterations in the function or expression of GLUT2 are involved in the Fanconi–Bickel syndrome, diabetes, and cancer. Distinguishing GLUT2 transport in tissues where other GLUTs coexist is challenging due to the low affinity of GLUT2 for glucose and fructose and the scarcity of GLUT-specific modulators. By combining in silico ligand screening of an inward-facing conformation model of GLUT2 and glucose uptake assays in a hexose transporter-deficient yeast strain, in which the GLUT1-5 can be expressed individually, we identified eleven new GLUT2 inhibitors (IC_50_ ranging from 0.61 to 19.3 µM). Among them, nine were GLUT2-selective, one inhibited GLUT1-4 (pan-Class I GLUT inhibitor), and another inhibited GLUT5 only. All these inhibitors dock to the substrate cavity periphery, close to the large cytosolic loop connecting the two transporter halves, outside the substrate-binding site. The GLUT2 inhibitors described here have various applications; GLUT2-specific inhibitors can serve as tools to examine the pathophysiological role of GLUT2 relative to other GLUTs, the pan-Class I GLUT inhibitor can block glucose entry in cancer cells, and the GLUT2/GLUT5 inhibitor can reduce the intestinal absorption of fructose to combat the harmful effects of a high-fructose diet.

## Introduction

Human glucose transporters (GLUTs), proteins of the *SLC2* gene family, facilitate the diffusion of hexoses into the cell and play a pivotal role in glucose homeostasis^1^. Important diseases, including cancer^2^ and diabetes^3,4^, are related to the dysfunction or misregulation of these transporters, identifying them as potential drug targets^1^. The 14 GLUT isoforms present in humans show an amino acid identity of 19–65% (homology of 42–81%)^5^ but differ in substrate specificity, affinity, and tissue distribution^6^. According to their sequence similarities, three classes of GLUTs have been defined^7^ with GLUT1-4 representing Class I, GLUTs 5, 7, 9, and 11 in Class II and GLUTs 6, 8, 10, 12, and 13 forming Class III^6,7^.

All GLUTs are furthermore part of the large major facilitator superfamily (MFS) and display the MFS-typical structure of twelve transmembrane helices (TM1–TM12; Supplementary Fig. S1) organized in two halves (N-terminal half: TM1–TM6 and C-terminal half: TM7–TM12) with a central substrate cavity^5^. Proposed mechanisms for sugar transport are the alternating access mechanism^8^, in which the substrate binding site is open to either the outside (exofacial, outward-facing) or inside (endofacial, inward-facing) space of the cell, and the fixed-site transporter with concurrent endo- and exo-facial substrate binding sites^9,10^. A hybrid mechanism that combines features of both proposed mechanisms, and is consistent with structural, kinetic, and modeling data of GLUT1 suggests that this transporter is an oligomer of allosteric, alternating access transporters^11^**.**

Three-dimensional structure determinations of GLUTs^12–14^ and their bacterial homologues^5,15^ have provided important information on the transport mechanisms and paved the way for structure-based drug design^16^, as exemplified by the discovery of a specific GLUT5 inhibitor^17^. For this, an extensive number of small compounds are screened in silico for binding to the respective transporter, as a first step^17^. Promising candidates can then be examined in an appropriate assay system^16^, and, if successful, be further developed into drugs.

Among GLUTs, GLUT2 is unique in its very low apparent affinity for glucose (K_M_ =  ~ 17 mM)^18^, which significantly surpasses fasting blood glucose levels (~ 5.5 mM)^19^, and for the substrates fructose (K_M_ =  ~ 76 mM)^6^, galactose (K_M_ =  ~ 92 mM)^6^, and mannose (K_M_ =  ~ 125 mM)^6^. The significance of GLUT2 lies in its regulatory functions such as glucose sensing^3^ and signaling^20,21^. Evidently, GLUT2 plays a crucial role in maintaining glucose homeostasis in many human tissues, such as the intestine, liver, kidney, and brain^20–25^.

Inactivating mutations in the GLUT2-encoding gene lead to the rare but severe Fanconi–Bickel syndrome^26^. Patients suffering from this autosomal, recessive disease show very diverse symptoms^26^, and its treatment is challenging due to the lack of effective drugs^27^. The wide spectrum of Fanconi–Bickel syndrome symptoms, including many atypical ones, also make its diagnosis difficult, and more cases with unusual pathological reports are still discovered^26^. Due to its many regulatory functions, a role of GLUT2 in the pathogenesis of diabetes is also discussed^28–30^. Generally, glucose sensing processes are complex and the full extent of GLUT2 in them is not yet fully elucidated; therefore, an abnormal function of GLUT2 might be the still undiagnosed cause of more clinical signs. Hence, GLUT2 is an important pharmaceutical target, and specific effectors will allow a more tailored treatment for GLUT2-involving diseases and expand our knowledge about its physiological role when applied in relevant studies. Furthermore, identifying GLUT2-specific ligands might provide new ways to explore the basis of substrate specificity among GLUT members^31^.

Several compounds were discovered that effectively inhibit glucose uptake via GLUTs in cell lines^32^. Glupin and glutor, for example, are very potent inhibitors for GLUT1 and 3 or GLUT1-3, respectively; 2-deoxy-glucose uptake experiments with human cell lines revealed their high potency with IC_50_ values in the nanomolar range^33,34^. Moreover, in presence of glupin or glutor the growth of several cancer cell lines was attenuated. Similarly, the pan-Class I inhibitor DRB18 showed potent anticancer activity^35^, supporting Class I GLUT inhibitors as promising anticancer probes. Several flavonoids are known to have an inhibitory effect on GLUT2, including quercetin, phloretin, isoquercitrin, myricetin, fisetin, apigenin, and tiliroside^36,37^. However, most show little potency (IC_50_ > 60 µM)^36^, and the more potent inhibitors quercetin and phloretin (IC_50_ < 4 µM)^38^ inhibit not only GLUT2 but also other GLUTs^38^ or, in the case of quercetin, the Vitamin C transporter SVCT1^39^. Identifying new, potent, and specific GLUT2 inhibitors is desirable but challenging due to GLUT2 low affinity for glucose and fructose and a background uptake of these substrates by other GLUT isoforms in human cell lines.

In this study, we used the recently established yeast cell-based system expressing human GLUT2^38^ to screen 163 small compounds that have been selected by in silico ligand screening of a GLUT2 inward-facing conformation model. Eleven candidates showed a high potency (IC_50_ < 20 µM) for inhibiting glucose uptake via GLUT2. Further examination of these GLUT2 inhibitors in the yeast cell-based systems expressing GLUT1^40^, GLUT3^38^, GLUT4^40^, or GLUT5^41^, showed that nine inhibitors were GLUT2-selective, one inhibited all Class I GLUTs but not GLUT5, and another inhibited GLUT5 but not Class I GLUTs. These candidates are a valuable addition to already existing GLUT2 inhibiting compounds. They will promote the development of GLUT-targeting drugs and a better understanding of GLUT2 biological role in health and disease.

## Results

### In silico ligand screening against GLUT2 inward-facing conformation structural model

Depending on which side of the cell membrane the substrate cavity opens to, GLUTs have two major conformations captured by the crystal structures of some isoforms and GLUT bacterial homologs^5,12–15^. For Class I GLUTs, inward-facing conformations have been determined for GLUT1^12^, and outward-facing conformations for GLUT3^13^; three-dimensional structures for GLUT2 and GLUT4 are not available. In silico ligand screening requires a structural model for the protein target. Even in the absence of crystal structures, the homology modeling of GLUTs based on the available crystal structures has been used successfully to identify new specific ligands. For instance, in silico ligand screening with a GLUT5 model in the inward-facing conformation, generated based on the bacterial GLUT homolog GlcP_Se_^5^, produced the first potent and specific GLUT5 inhibitor^17^.

**Figure 1:**
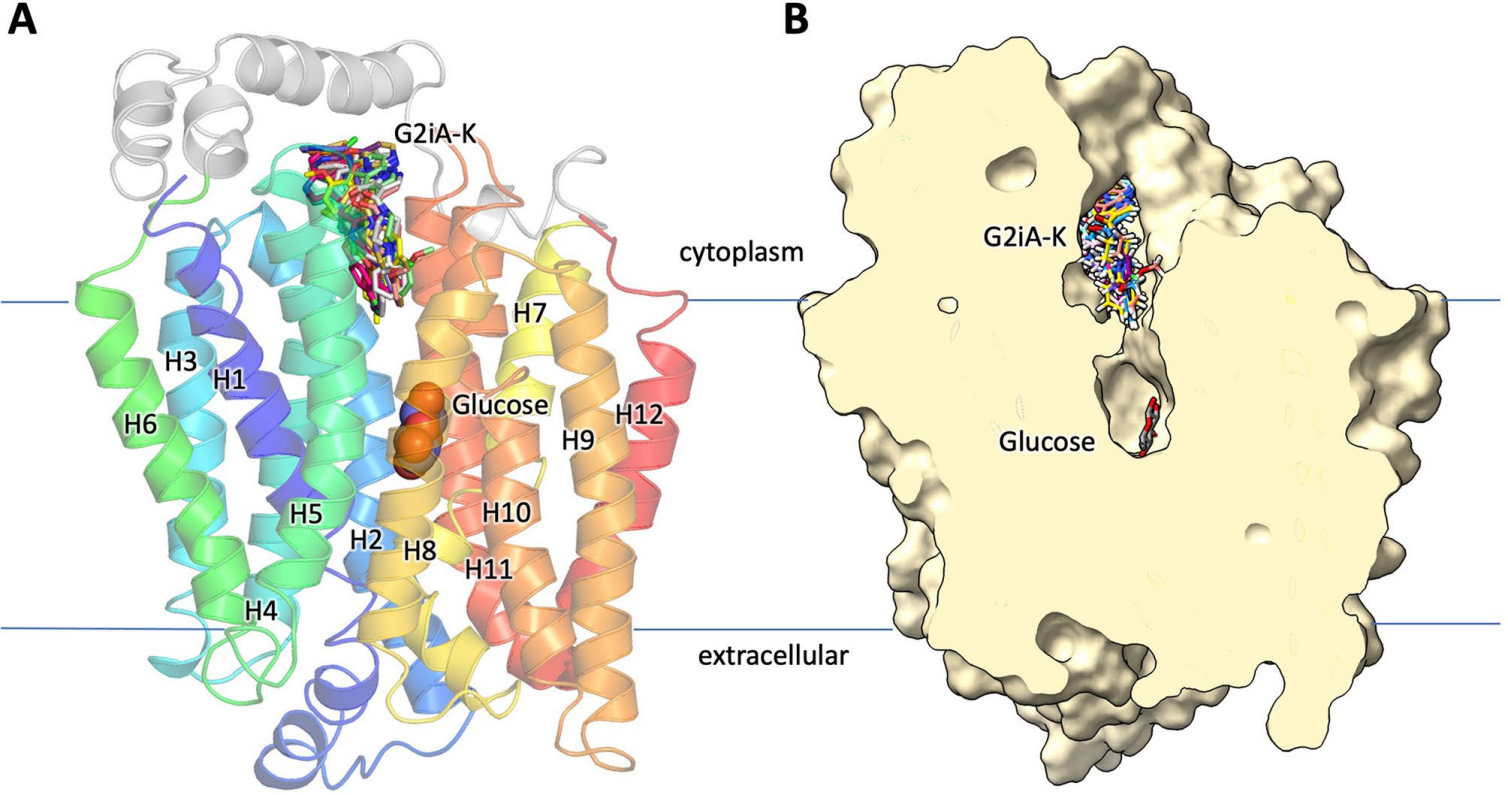
GLUT2 inward-facing conformation model and selected ligands from the virtual screening. The inward-facing conformation of GLUT2 showing the glucose binding site (glucose shown in sphere representation) and several ligands from the virtual screening (Table 1) bound above the glucose binding site (ligands shown as stick representation, in different colors). The homology model was generated based on the GLUT1 crystal structure (PDB ID: 4PYP). (**A**) GLUT2 model in ribbon diagram, with the transmembrane helices color-coded from blue (first transmembrane helix, H1) to red (last transmembrane helix, H12) (see also Fig. 4L). (**B**) Central slice through the GLUT2 isosurface showing the substrate cavity containing the glucose binding site and docked ligands. The figure was generated with ChimeraX Version 1.2 (https://www.rbvi.ucsf.edu/chimerax/) and PyMOL Version 2.3.0 (https://pymol.org/).

The structural model for the GLUT2 inward-facing conformation (Fig. 1) was modeled based on the crystal structure of GLUT1 (PDB ID: 4PYP) with the Molecular Operating Environment (MOE) software (https://www.chemcomp.com/). GLUT1 and GLUT2 share 52% and 68% protein sequence identity and similarity, respectively, as determined with Align function in MOE. The docking site, containing the substrate cavity without the substrate binding site, was prepared using OpenEye FRED software (https://www.eyesopen.com/). The ChemNavigator library of over 6 million commercially available compounds was prepared for docking using Omega2 and FRED software (https://www.eyesopen.com/). The docking studies were conducted using OpenEye FRED software. Docked compounds were scored using Chemgauss4 scoring function. The compounds docked in sites distinct from that of glucose, closer to the substrate cavity entrance (Fig. 1B). Considering commercial availability and affordability, we purchased 163 out of the top 200 scored compounds for experimental validation.

### Screening of the lead candidates in the GLUT2-expressing ***hxt***^0^ yeast system

To test the 163 compounds selected by in silico ligand screening, we utilized the *hxt*^0^ (hexose transporter-deficient) yeast system that expresses human GLUT2^38^. Similar GLUT-specific *hxt*^0^ yeast systems are available for all Class I GLUTs and GLUT5^38,40–42^, providing a convenient assay platform for these transporters’ ligands^16^. For GLUT2, the applied yeast strain EBY.S7 is devoid of all its endogenous hexose transporters (*hxt*^0^) and carries the *fgy1* mutation^36^ in the *EFR3* gene, proven to be beneficial for the heterologous expression of human GLUTs^16^. The active expression of the transporter required a GLUT2 version with a truncated loop between transmembrane regions TM1 and TM2 and an additional point mutation (GLUT2_∆loopS_Q455R_)^38^. GLUT2_∆loopS_Q455R_ recapitulates the functional properties of GLUT2 closely. For example, GLUT2 expressed in *Xenopus laevis* oocytes had K_M, Glucose_ = 17 mM^18^ and K_M, Fructose_ = 66.7 ± 18.3 mM^43^, while GLUT2_∆loopS_Q455R_ had K_M, Glucose_ = 14.1 ± 1.3 mM and K_M, Fructose_ = 87.0 ± 8.2 mM (Supplementary Fig. S2). Also, reported GLUT2 inhibitors, phloretin and quercetin^36^, inhibited similarly GLUT2_∆loopS_Q455R_^38^, confirming this system’s applicability to screening GLUT2 inhibitors.

**Figure 2:**
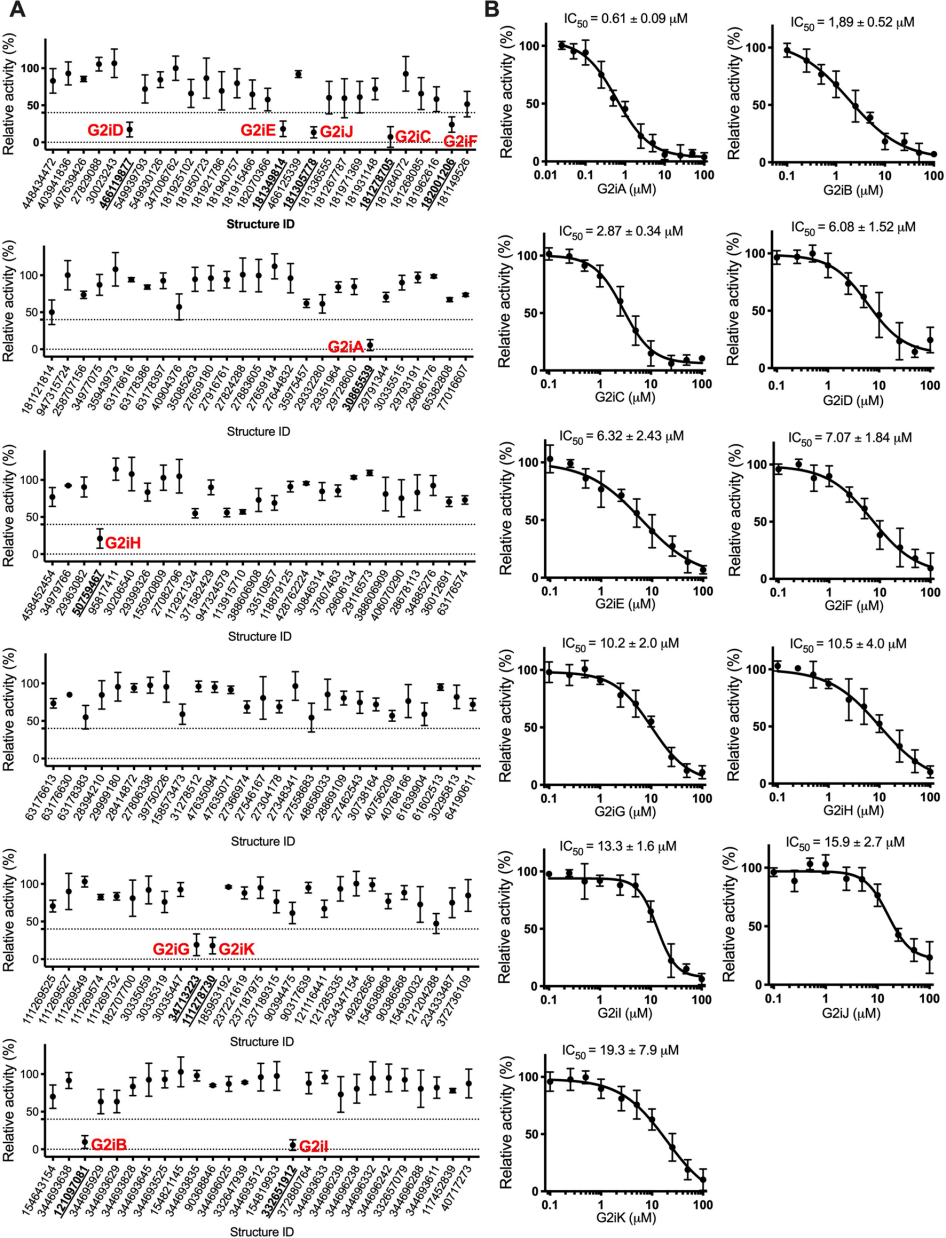
Effect of compounds identified from in silico ligand screening on GLUT2 transport activity. (**A**) GLUT2 relative transport activity at 15 mM glucose concentration, in the presence of 100 µM compound concentration (see “Materials and methods” for details). The compounds (see also Supplementary Table S1) are identified by the ChemNavigator structure ID. Eleven compounds (designated as G2iA-K, in red, with the corresponding structure ID in bold, underlined font) inhibited GLUT2 relative activity by more than 60% (marked by the dotted line). (**B**) Dose–response curves for G2iA-G2iK (see also Table 1) inhibition of GLUT2 transport activity. Standard deviations for experimental points, represented by error bars, come from at least three independent measurements.

GLUT2 transport activity was determined as previously described^38^. Pre-grown yeast cells were washed and resuspended in PBS buffer to an OD_600nm_ of ~ 10; 100 µl of this cell suspension constituted the assay mix. Uptake activity of GLUT2 was determined by adding C^14^-hexose (glucose or fructose), quenching after 10 min, filtering the cells, and measuring the radioactivity with a scintillation counter. Initial compound screening for GLUT2 inhibition was performed at 15 mM glucose concentration (i.e., ~ K_M_) and 100 µM of each chemical. While none of the tested compounds mediated an increase in glucose uptake activity by GLUT2, several diminished it significantly (Fig. 2A). Among these, 11 compounds decreased GLUT2 activity by at least 60% and were further examined to determine their respective IC_50_ value (Fig. 2B). All compounds are effective inhibitors (IC_50_ < 20 µM); for simplicity, we named them G2i (from GLUT2 inhibitor) A–K in the order of decreasing inhibition potency (Table 1, Fig. 2). G2iA showed the strongest GLUT2 inhibition with an IC_50_ of 0.61 µM, almost twice as strong as phloretin and five times more potent than quercetin^38^.

**Table 1:** Structures and chemical names of potent (IC_50_ < 20 µM) GLUT2 inhibitors. Structure_ID refers to the ChemNavigator structure identifier.

GLUT2 inhibitor	Structure_ID	Chemical name	Structure	IC_50_ (µM)
G2iA	30865539	4-(5-(4-Fluorophenyl)-1-{[(2-methyl-1H-indol-3-yl)sulfanyl]acetyl}-4,5-dihydro-1H-pyrazol-3-yl)phenyl methyl ether		0.61 ± 0.09
G2iB	121097081	*N*-Benzyl-*N*-(2-{[4-(4-chlorophenyl)-1-(3,4-dimethoxyphenyl)-1H-imidazol-2-yl]amino}-2-oxoethyl)-4-methylbenzamide		1.89 ± 0.52
G2iC	181278705	2-(5-Cyclopropyl-4-{[4-(2-methoxyphenyl)-1-piperazinyl]carbonyl}-1H-pyrazol-1-yl)-4-(2-thienyl)pyrimidine		2.87 ± 0.34
G2iD	466119877	2-{5-(Methoxymethyl)-4-[(4-phenyl-1-piperazinyl)carbonyl]-1H-pyrazol-1-yl}-6,7-dihydro-5H-benzo[6,7]cyclohepta[1,2-d]pyrimidine		6.08 ± 1.52
G2iE	181349814	2-(4-(1-Benzothien-3-yl)-2-{[4-(2-pyridinyl)-1-piperazinyl]methyl}phenoxy)-*N*-(1,3-thiazol-2-ylmethyl)acetamide		6.32 ± 2.43
G2iF	182001206	5-Cyclopropyl-1-(5,6-dihydrobenzo[h]quinazolin-2-yl)-*N*-methyl-*N*-(5-quinolinylmethyl)-1H-pyrazole-4-carboxamide		7.07 ± 1.84
G2iG	34713223	*N*-(4-Isopropylphenyl)-3-{[4-(2-methoxyphenyl)-1-piperazinyl]carbonyl}-4-oxo-1,4-dihydro-6-quinolinesulfonamide		10.2 ± 2.0
G2iH	50759467	*N*-[2-(2-Chlorophenyl)-2-(1H-indol-3-yl)ethyl]-2-(1H-indol-3-yl)acetamide		10.5 ± 4.0
G2iI	332651912	3-(5-Chloro-1H-indol-3-yl)-3-[3-(4-chlorophenoxy)phenyl]-*N*-[2-(4-morpholinyl)ethyl]propanamide		13.3 ± 1.6
G2iJ	181305778	2-(5-Cyclopropyl-4-{[4-(2-ethoxyphenyl)-1-piperazinyl]carbonyl}-1H-pyrazol-1-yl)-4-(5-methyl-2-furyl)pyrimidine		15.9 ± 2.7
G2iK	111278730	1-(5,6-Dimethylfuro[2,3-d]pyrimidin-4-yl)-*N*-[2-(5-methyl-1H-indol-3-yl)ethyl]-3-piperidinecarboxamide		19.3 ± 7.9

### Effect of GLUT2 inhibitors on the other Class I GLUTs and GLUT5

**Figure 3:**
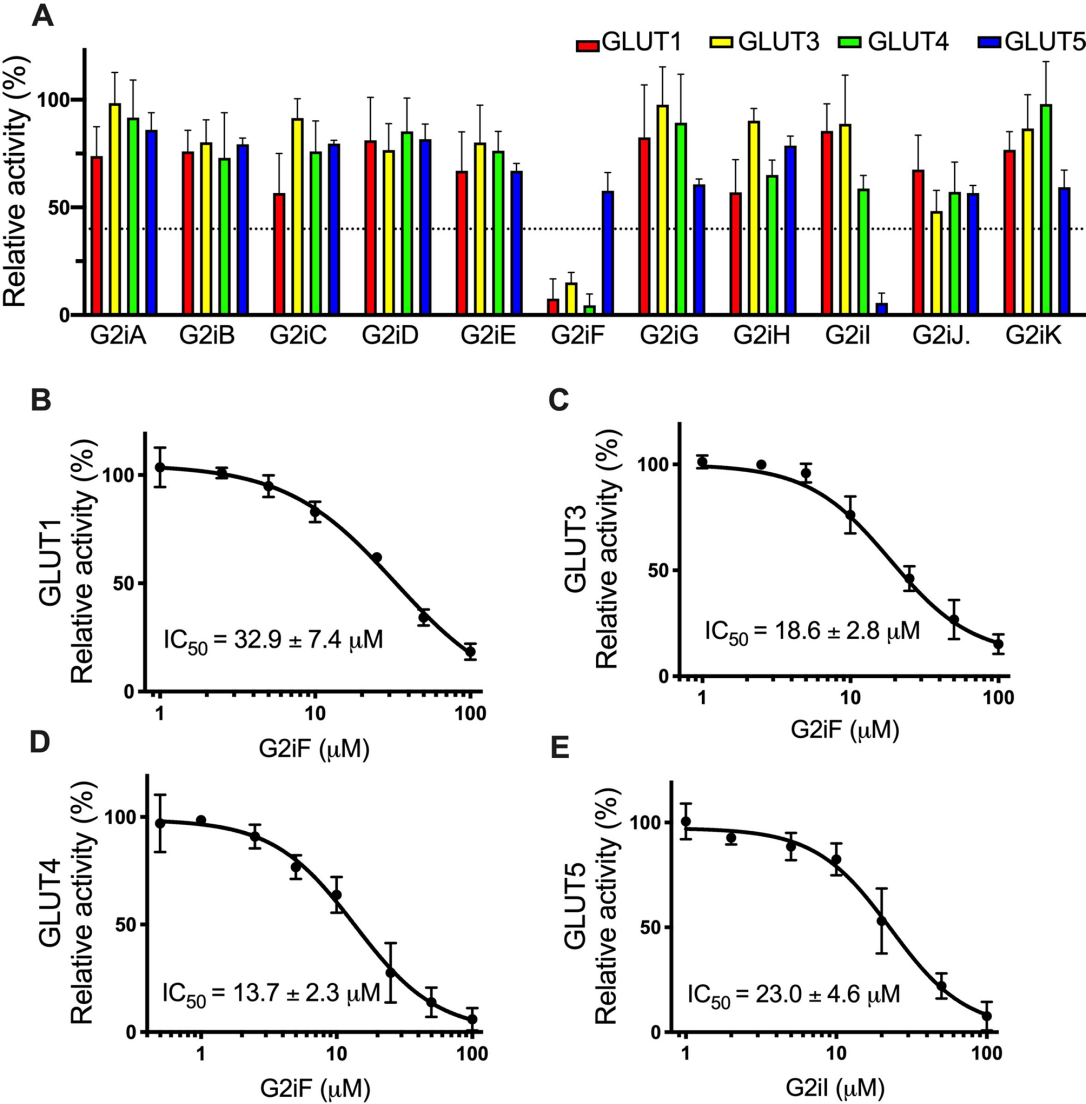
Effect of GLUT2 inhibitors on GLUT1, GLUT3, GLUT4, and GLUT5. (**A**) Relative transport activity of GLUT1 (red), GLUT3 (yellow), GLUT4 (green), and GLUT5 (blue) in the presence of 100 µM of GLUT2 inhibitors G2iA-G2iK. Dose–response curves for G2iF inhibition in GLUT1 (**B**), GLUT3 (**C**), and GLUT4 (**D**), and G2iI inhibition in GLUT5 (**E**). Substrate conditions for relative transport activity were: 1.5 mM glucose for GLUT3, 5 mM glucose for GLUT1 and GLUT4, and 10 mM fructose for GLUT5. Error bars show standard deviation from at least three independent measurements.

Establishing the selectivity of GLUT2 inhibitors for other GLUT isoforms, particularly its closely related Class I GLUTs, is crucial for future application of these inhibitors. Often several GLUTs coexist in the same tissue, and being able to modulate selectively an individual GLUT provides a powerful tool in unraveling its pathophysiological role. Therefore, to determine the selectivity of the identified GLUT2 inhibitors, we tested them for their effect on the GLUT homologs GLUT1, 3, 4, and 5. For this, *hxt*^0^ yeast cells actively expressing the respective transporter^38,40–42^ were incubated with 100 µM of the tested compound, and the transport activity was assayed in the same manner as for GLUT2 but at substrate concentrations close to the K_M_ in the respective GLUT (i.e., 5 mM glucose for GLUT1^44^ and GLUT4^44^, 1.5 mM glucose for GLUT3^43^, 10 mM fructose for GLUT5^41^) (Fig. 3A). GLUT2 is more closely related to the other Class I GLUTs (52–65% sequence identity) than GLUT5 (Class II GLUT, 40% sequence identity)^5^. Nevertheless, most GLUT2 inhibitors seem to have only negligible inhibitory effects on the other GLUTs (Fig. 3A). Thus, only G2iF inhibits GLUT1, 3, and 4, whereas G2iI decreased just GLUT5 activity (Fig. 3A). However, G2iF IC_50_ values were higher for other GLUTs (33 µM for GLUT1, 19 µM for GLUT3 and 14 µM for GLUT4) than for GLUT2 (7 µM); the same was found for the IC_50_ of G2iI (23 µM for GLUT5 vs. 13 µM for GLUT2) (Fig. 3B–E). Importantly, all other tested compounds, including the most potent GLUT2 inhibitor G2iA appear not to significantly affect the other GLUTs tested, indicating that these are GLUT2-specific.

### Docking sites of GLUT2 inhibitors

The virtual ligand screening showed that all 11 GLUT2 inhibitors docked to the inward-facing conformation of GLUT2 in sites distinct from that of glucose, closer to the substrate cavity entrance (Fig. 1B). The two most potent GLUT2 inhibitors, G2iA and G2iB, showed noncompetitive inhibition with glucose (Supplementary Fig. S3), consistent with their binding site being distinct from that of the substrate.

**Figure 4:**
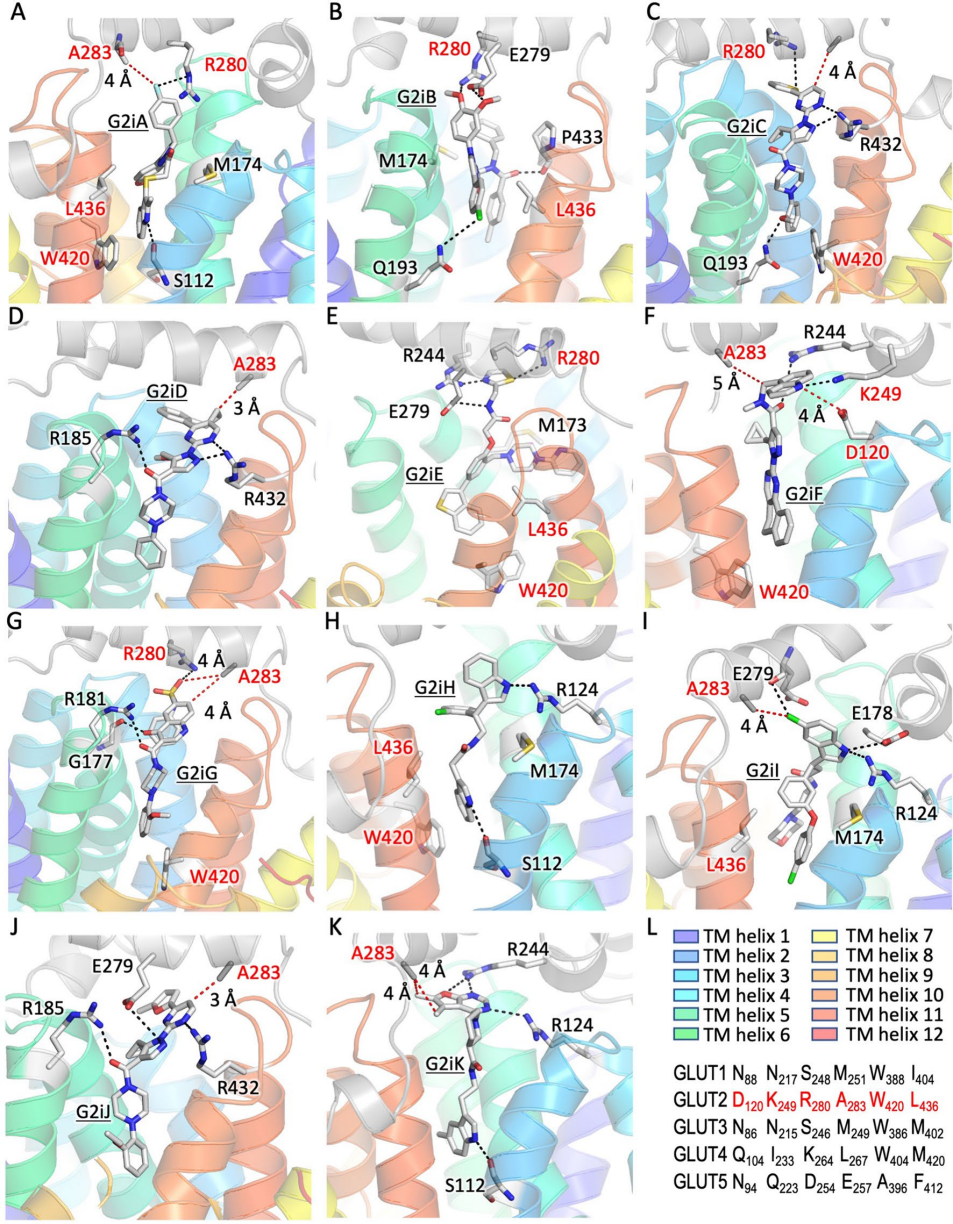
Docking sites of G2iA-G2iK in the inward-facing conformation GLUT2 model. G2iA-G2iK dock in the superior region of the substrate cavity, distinct from the glucose binding site (see Fig. 1B). Close-up views for the docked ligands: G2iA (**A**), G2iB (**B**), G2iC (**C**), G2iD (**D**), G2iE (**E**), G2iF (**F**), G2iG (**G**), G2iH (**H**), G2iI (**I**), G2iJ (**J**), and G2iK (**K**). Residues that are not conserved in GLUT1-5 are in red. (**L**) GLUT1-5 sequence alignment for unconserved protein residues interacting with G2iA-G2iK in the GLUT2 model. The color-code identification of transmembrane (TM) helices is the same as in Fig. 1A.

Protein–ligand interactions (Fig. 4 and Supplementary Fig. S4) include hydrogen-bonds with charged residues from the cytosolic loops or transmembrane (TM) helix ends (D120, R124, E178, R181, R185, R244, K249, E279, R280, R432), backbone carbonyls (G177, P433) or polar residues from TM helices (S112, Q193); hydrophobic or Van der Waals interactions (M174, A283, L436); and cation-pi interactions with guanidinium groups (R244, R280). Among these, the residues that are not conserved in GLUT1-5 are D120, K249, R280, A283, W420, and L436 (Fig. 4L). G2iA is oriented in its pocket by hydrophobic interactions with M174 and L436, a hydrogen bond of its amino indole group with the sidechain of S112, a polar interaction of its phenyl fluorine with the R280 guanidinium group, as well as a cation-pi interaction of the fluorophenyl group with the R280 sidechain (Fig. 4A). Hydrophobic interactions with M174 and L436 also contribute to the pockets of G2iB (Fig. 4B), G2iE (Fig. 4E), G2iH (Fig. 4H), and G2iI (Fig. 4I). R280 sidechain makes hydrogen bond interactions with oxygens from the methoxyl group of G2iB (Fig. 4B) or the sulfamide group of G2iG (Fig. 4G), and the sulfurs of the G2iC thienyl group (Fig. 4C) or of the G2E thiazol group (Fig. 4E). It also has cation-pi interaction with the G2iC thienyl group and G2iF quinoline moiety. The α-carbon of A283 comes close (3 Å) to G2iD (Fig. 4D); this inhibitor has Van der Waals interactions with the large cytosolic loop. In G2iF, besides the cation-pi interaction with the quinoline, R244 also makes a hydrogen bond with the ligand’s carbonyl, suggesting that positioning of R244 is essential for G2iF recognition.

## Discussion

GLUT2 shares characteristic motifs and high similarity with the other Class I representatives GLUTs 1, 3, and 4^7^. In this group, it is the only transporter that also accepts fructose as a substrate^6^. This prompted us to test the identified GLUT2-inhibiting compounds against all Class I GLUTs and the Class II member GLUT5, a fructose-only transporter, to elucidate their specificity. Except for G2iF, which also inhibits other Class I GLUTs (although to a lesser extent: IC_50_ in GLUT2 = 7 µM, IC_50_ in other Class I GLUTs ≥ 14 µM), and G2iI which inhibits GLUT5 (IC_50_ = 23 µM) less potently than GLUT2 (IC_50_ = 13 µM), the other nine GLUT2 ligands did not show a significant effect on the tested transporters (Fig. 3). Although so far unknown effects on other human transporters cannot be ruled out completely, these data indicate that, for the first time, very potent and specific GLUT2 inhibitors were identified. For example, similarity search (Tanimoto >  = 0.9, across the entire database, all the other options default) in PubChem for G2iA and G2iB returned 60 compounds with 34 bioactivity records and 409 compounds with 1 bioactivity record, respectively. The bioactivity records for all similar compounds are labeled as inactive. Identical searches with the exact similarity cutoffs, when performed on the ChEMBL database (https://www.ebi.ac.uk/chembl/), found no similar compounds. These results indicate that, based on current knowledge collated in two of the largest public databases, G2iA and G2iB are potentially selective for GLUT2 and can become valuable tools for probing and understanding GLUT2 biology.

A possible explanation for the high prevalence of GLUT2-selective inhibitors among the identified GLUT2 inhibitors may be that the inhibitors target the upper portion of the substrate cavity. All 11 inhibitors dock at the substrate cavity entrance, separately from the glucose binding site (Figs. 1, 4). As noted above, G2iA and G2iB are noncompetitive with glucose, confirming that the inhibitors bind at a different site than the substrate (Supplementary Fig. S3). The substrate cavity base containing the glucose binding site is made up of residues mostly conserved in Class I GLUTs (Supplementary Fig. S1A). The cytosolic entrance of the substrate cavity surrounded by soluble loops, especially the large loop connecting the N- and C-domains of the transporter (GLUT2 amino acid residues 240 -298), has much more variability in the protein sequence (Supplementary Fig. S1). Thus, the binding site location of the GLUT2 inhibitors is consistent with the GLUT2 selectivity exhibited by most of these ligands and suggests that targeting the entrance of the substrate cavity, whether in the inward- or outward-facing conformations, for ligand screening may increase the chances of producing GLUT-specific ligands. Also, given the separation between glucose and such inhibitor sites, bridging the two sites by attaching a glucosyl group to these types of inhibitors may substantially improve inhibitor potency while maintaining selectivity. Additionally, such compounds could help to crystallize GLUTs whose structures are yet unknown, including GLUT2, as the combination of substrate and inhibitor would greatly stabilize the transporter conformation.

A significant difference in the substrate sites between Class I GLUTs and GLUT5 is W420_GLUT2_ (W388_GLUT1_), conserved in Class I GLUTs but replaced by a smaller residue in Class II GLUTs (e.g., A396 in GLUT5). This substitution creates more space in the substrate cavity, changing the binding mode of ligands and substrate specificity^31^. For instance, GLUT5_A396W_ mutant became a transporter of both glucose and fructose, while the wild-type can only transport fructose. Therefore, it is likely that GLUT2 inhibitors adopt different binding modes in GLUT5 than those described for GLUT2.

Analysis of the docked GLUT2 inhibitors from the virtual ligand screening (Fig. 4) suggests that L436, R280, A283—residues not conserved in other Class I GLUTs or GLUT5—may play a role in the selectivity of GLUT2 inhibitors. The equivalent substitutions of R280 in other GLUTs (S248_GLUT1_, S246_GLUT3_, K264_GLUT4_, D254_GLUT5_) would decrease or abolish this residue’s interactions with G2iA, G2iB, G2iC, G2iE, and G2iG (Fig. 4). A bulkier side chain in the position of A283 (M251_GLUT1_, M249_GLUT3_, L267_GLUT4_, E257_GLUT5_) could sterically interfere with the binding of G2iD, G2iG, G2iI, G2iJ, and G2iK. L436 (I404_GLUT1_, M402_GLUT3_, M420_GLUT4_, F412_GLUT5_) may be important in shaping the hydrophobic interactions in the binding pockets for G2iA, G2iB, G2iE, G2iH, and G2iI. The recognition of G2iF, the pan-Class I GLUT inhibitor, relies mostly on R244, a conserved residue in GLUT1-5. This sidechain has hydrogen bond and cation-pi interactions with G2iF, suggesting that the guanidinium group’s position is critical. The quinoline nitrogen of G2iF makes a hydrogen bond with K249 (N217_GLUT1_, N215_GLUT3_, I233_GLUT4_, Q223_GLUT5_) and a weak interaction with D120 (N88_GLUT1_, N86_GLUT3_, Q104_GLUT4_, N94_GLUT5_). The equivalent substitutions in these positions for GLUT1-4 are still able to maintain interactions with G2iF. In GLUT4, the substitution of D120 with a glutamine residue would result in better interaction with G2iF, consistent with the lower IC_50_ of this inhibitor for GLUT4, relative to the other Class I GLUTs (Fig. 3).

With this study, we present a range of molecules that will serve as valuable tools to investigate the physiological role of GLUT2 in health and disease and may evolve to therapeutic drugs in GLUT2-related diseases. Given their selectivity against other hexose transporters, we believe these compounds could serve as chemical probes for the in-depth study of GLUT2. Indeed, GLUT2 may play a role in several important diseases^27,28,45,46^. It is upregulated in several cancer types like pancreatic, hepatic, micropapillary, or colon cancer^47^. Inhibition of GLUT2 via the non-specific inhibitor phloretin has been shown to diminish tumor growth in colon cancer^48^ and hepatocellular carcinoma^49^. The Class I GLUTs 1 and 3 are also overexpressed in many cancer types and related to elevated tumor growth and poor survival^50^. For cancer treatment, the non-specific inhibitor G2iF that inhibits Class I GLUTs but not GLUT5 might join phloretin as a putative drug^49^. Furthermore, substantial overexpression of the fructose transporters GLUT2 and GLUT5 lead to the hypothesis that certain cancer cells use fructose as a preferential carbon source^47^. In these cases, the here presented GLUT2/GLUT5 inhibiting compound G2iI might be a promising candidate in the combat against cancer and other high-fructose diet-related diseases^51^. Importantly, a potent and GLUT2-specific effector (e.g., G2iA) might further elucidate the particular role of GLUT2 in tumor pathogenesis and facilitate studies targeting GLUT2, thereby contributing to unravel complex cancer behavior further.

In healthy individuals, GLUT2 traffics to the apical side of the brush border membrane only after a meal, when glucose concentrations in the lumen are high, to support SGLT1 and accelerate glucose uptake^28^. In morbidly obese humans, a consistent location of GLUT2 at the apical membrane, even in fasting states, was observed and related to insulin resistance^52^. This might result in higher glucose levels in the lumen in fasting states and an abnormal sugar supply could support bacterial growth which interferes with a healthy gut microbiome^52^. Specific inhibition of GLUT2 could mitigate such pathologies. An altered microbiome composition in mice with intestinal-specific GLUT2 deletion has been detected in previous studies^53^, supporting the gut microbiome as a possible field of application for GLUT2 inhibitors. Also, Schmitt et al. showed that GLUT2 deletion in the murine intestine causes favorable effects like improved glucose tolerance and diminished body weight gain^53^. This suggests that GLUT2 tailored inhibitors could lead to similar results and might be applied in morbidly obese patients or type 2 diabetic persons with beneficial health effects.

Interestingly, viral infections affect the expression of GLUT2. While the hepatitis C virus downregulates GLUT2 expression^54^, the transmissible gastroenteritis virus upregulates the transporter’s expression, enhancing intestinal glucose absorption, which promotes viral replication^55^. Hence, GLUT2 inhibition could assist in the containment of certain viruses. Clearly, the role of GLUT2 in the metabolic processes is highly complex and not fully understood. Therefore, the application of GLUT2-specific inhibitors also bears high risks as it might have not only beneficial but also adverse effects, and more studies are necessary to increase our level of knowledge. However, accessibility of specific GLUT2 inhibitors represents a tremendous advantage over less-specific GLUT inhibitors in developing drugs with a defined effective spectrum and lower side effects.

These compounds are valuable tools in the efforts of answering many open questions concerning GLUT2. For instance, it is still unclear how GLUT2 is mobilized in response to glucose in various cell types and different pathologies^56^. Possible players include the type of membrane lipids^57^, protein partners^56,58^, or glycosylation^3^. Distinct from other GLUTs, the extraordinary low affinity for glucose and fructose probably assigns special functions of glucose sensing^59^ and signaling^21^ to GLUT2, but the detailed molecular functions remain to be elucidated. Furthermore, the Fanconi–Bickel syndrome due to GLUT2 malfunction^27^ has various symptoms that indicate yet undiscovered physiological roles for GLUT2, and the transporter’s role in certain cancer types remains unclear^47^. Future studies will benefit from the existence of a range of easily accessible GLUT2-specific inhibitors with varying affinities.

## Materials and methods

Yeast plasmids and *hxt*^0^ strains were from Dr. Mislav Oreb and Dr. Eckhard Boles (Goethe University Frankfurt, Germany). The tested compounds were purchased from MilliporeSigma (St. Louis, MO, USA).

### In silico ligand screening

GLUT2 homology models were build using Molecular Operating Environment (MOE) software (www.chemcomp.com). Based on sequence alignment between GLUT1 and GLUT2, with the crystal structure for GLUT1 inward-facing conformation (PDB ID: 4PYP) as a template, the initial model geometry was generated, followed by refinement of the sidechains and energy minimization with the MMFF94x force field. The model with the lowest interaction energy and RMSD was selected for docking studies. Molecular probing of inner cavities was done to identify potential binding sites. Two sites of interest were identified in the proximity of both ends of the transmembrane regions and used for receptor preparation with OpenEye FRED software^60^ (https://www.eyesopen.com).

ChemNavigator collection (MilliporeSigma, St. Louis, MO, USA) of commercially available compounds (~ 6 million) was processed for docking studies using the following protocol: (i) remove all compounds that are not small organic molecules, (ii) remove salts counterions, (iii) normalize charges and select the most likely tautomer at pH 7, (iv) generate an ensemble of up to 400 molecular conformers for each compound using Omega2 software (https://www.eyesopen.com).

After completing the preparation steps, the virtual docking screen was performed with OpenEye FRED software on a Linux cluster. All conformer ensembles were docked into the selected sites described above, retaining only the best scoring pose based on the Chemgauss4 score for each compound. The top 200 best scoring compounds were extracted and selected for purchase and experimental validation. Due to availability and affordability issues, only 163 compounds were sourced and submitted for experimental validation.

### Culturing of GLUT-expressing ***hxt***^***0***^ yeast cells for transport assay

Depending on the plasmid selection marker, the media for cell culturing was either YEP [1% (w/v) yeast extract and 2% (w/v) peptone] supplemented with 100 µg/ml geneticin (G418) or complete synthetic media without uracil (SC-uracil). Yeast cell culturing was done at 30 ºC with shaking (180–220 rpm). The plasmids containing the functional constructs of GLUT1-5 (GLUT1, GLUT2_∆loopS_Q455R_, GLUT3_S66Y_, GLUT4, GLUT5_S72Y_) were transformed in the corresponding *hxt*^0^ strains (EBY.VW4000 for GLUT5, EBY.S7 for GLUT1-3, and EBY.S7 *Δerg4* for GLUT4)^38,40–42^ and grown on 2% (w/v) agar plates of the respective media supplemented with 1% (w/v) maltose. An initial culture of ~ 10 ml was started with a few colonies and grown for 2–3 days if the media was SC-uracil with 1% (w/v) maltose (for GLUT1, GLUT3, and GLUT4) or 1–2 days if the media was YEP with 1% (w/v) maltose and 100 µg/ml G418 (for GLUT2 and GLUT5). Cells were washed once in the corresponding media in which maltose was substituted with 0.1–2% (w/v) hexose substrate for the expressing GLUT (i.e., SC-uracil, 2% (w/v) glucose for GLUT1; SC-uracil, 0.2% (w/v) glucose for GLUT3 and GLUT4; YEP, 0.2% (w/v) glucose, 100 µg/ml G418 for GLUT2; and YEP, 2% (w/v) fructose, 200 µg/ml G418 for GLUT5). Then, cells were transferred in the same media to OD_600nm_ ~ 0.5 and grown further for 1–2 days.

### GLUT transport assay

Commercial providers for chemicals tested for GLUT2 inhibition are listed in Supplementary Table S1. C^14^-fructose and -glucose were from Moravek Inc (Brea, CA, USA). For transport activity assay, cells in the hexose media were centrifuged (1000×*g*, 5 min, room temperature), washed once with PBS buffer (10 mM Na_2_HPO_4_, 1.8 mM KH_2_PO_4_, 2.7 mM KCl, 137 mM NaCl, pH 7.4), and resuspended in PBS buffer at an OD_600nm_ ~ 10; each assay contained 100 µl of this cell solution. The transport activity assay was started by adding C^14^-hexose (5 mM glucose for GLUT1 or GLUT4, 1.5 mM glucose for GLUT3, 10 mM glucose for GLUT2, and 10 mM fructose for GLUT5). When determining the K_M_ for fructose and glucose in GLUT2, substrate concentrations were varied accordingly. Transport activity assay was stopped after 10 min by adding 3 ml ice-chilled Quench buffer (0.1 M KPi, 0.1 M LiCl, pH 5.5), followed by filtration through a glass fiber channel (GC50; Advantec, Tokyo, Japan) under vacuum and another wash with 3 ml Quench buffer and filtration. The filtration membranes were transferred into scintillation vials, combined with 10 ml of Scintillation Solution (BioSafeII; Research Products International, Mount Prospect, IL, USA), and vortexed briefly. The radioactivity was determined with a scintillation counter (Tri-carb 2900TR, Perkin Elmer, USA). The compounds were dissolved in dimethyl sulfoxide (DMSO) at 100× (i.e., ~ 10 mM) the final assay concentration. Controls for determining the relative transport activity included 1% (v/v) DMSO, representing the normal GLUT2 activity (100%), and known inhibitors 200 µM phloretin for GLUT1-4^38,44^, and 100 µM N-[4-(methylsulfonyl)-2-nitrophenyl]-1,3-benzodioxol-5-amine (MSNBA) for GLUT5^17^, representing fully inhibited activity. Primary screening was done at 100 µM compound concentration (see Supplementary Table S1 for a list of all tested compounds). The IC_50_ values were further determined for the compounds that diminished the relative transport activity by at least 60%. When determining the inhibition mode for the most potent GLUT2 inhibitors (IC_50_ < 2 µM), G2iA and G2iB, transport activity at 7, 15, and 30 mM glucose concentrations were determined in the absence or presence of different inhibitor concentrations (0, 0.66 and 2 µM for G2iA or 0, 1.66, 5 µM for G2iB; Supplementary Fig. S3). Data were analyzed with GraphPad Prism (San Diego, CA, USA).

## Supplementary Information

Supplementary Information.
