# Supplementary material for: Identification of new GLUT2-selective inhibitors through in silico ligand screening and validation in eukaryotic expression systems
Source: Sci Rep. 2021 Jul 2;11:13751. doi: 10.1038/s41598-021-93063-5 (PMC8253845; doi:10.1038/s41598-021-93063-5)
Supplement: Supplementary file 1 — Supplementary Information. [file 41598_2021_93063_MOESM1_ESM.docx]

**SUPPLEMENTARY INFORMATION**

**Identification of new GLUT2-selective inhibitors through *in silico* ligand screening and validation in eukaryotic expression systems**

Sina Schmidl^1,#^, Oleg Ursu^2,3,#^, Cristina V. Iancu^4^, Mislav Oreb^1^,

Tudor Oprea^2,5*^and Jun-yong Choe^4,6,*^

^1^ Institute of Molecular Biosciences, Faculty of Biological Sciences, Goethe University Frankfurt, Frankfurt am Main, Germany.

^2^ Translational Informatics Division, Department of Internal Medicine, The University of New Mexico School of Medicine, Albuquerque, NM 87131, USA.

^3^ Present address: Computational and Structural Chemistry, Merck & Co., Inc., 2000 Galloping Hill Road, Kenilworth, NJ 07033, USA.

^4^ Department of Chemistry, East Carolina Diabetes and Obesity Institute, East Carolina University, Greenville, NC 27834 USA.

^5^ UNM Comprehensive Cancer Center, The University of New Mexico, Albuquerque, NM 87131, USA.

^6^ Department of Biochemistry and Molecular Biology, The Chicago Medical School, Rosalind Franklin University of Medicine and Science, North Chicago, IL 60064 USA.

^#^ These authors contributed equally.

*Corresponding authors: Jun-yong Choe, E-mail: choej18@ecu.edu

Tudor Oprea, E-mail: toprea@salud.unm.edu

Supplementary Figure S1

Supplementary Figure S2

Supplementary Figure S3

Supplementary Figure S4

Supplementary Table S1


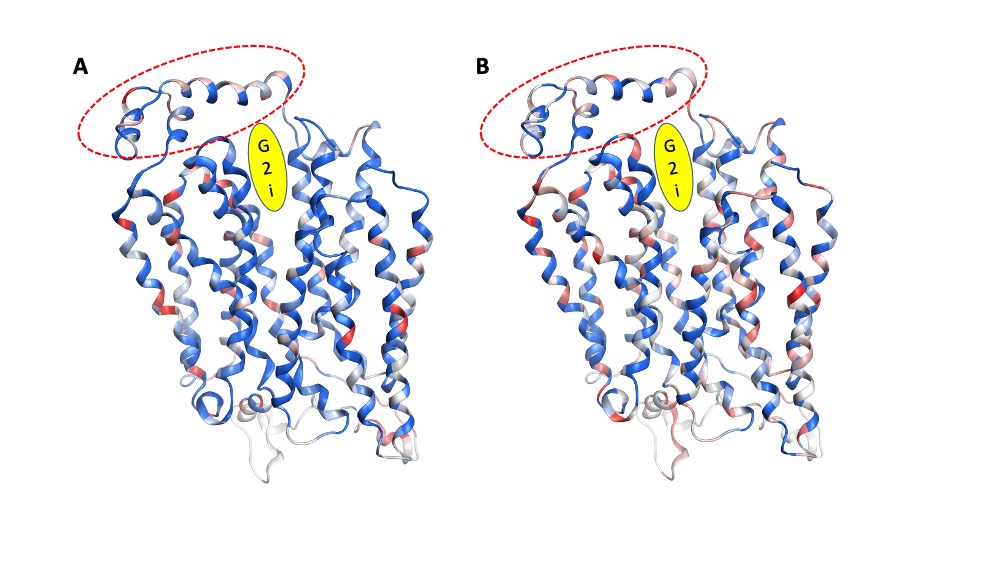


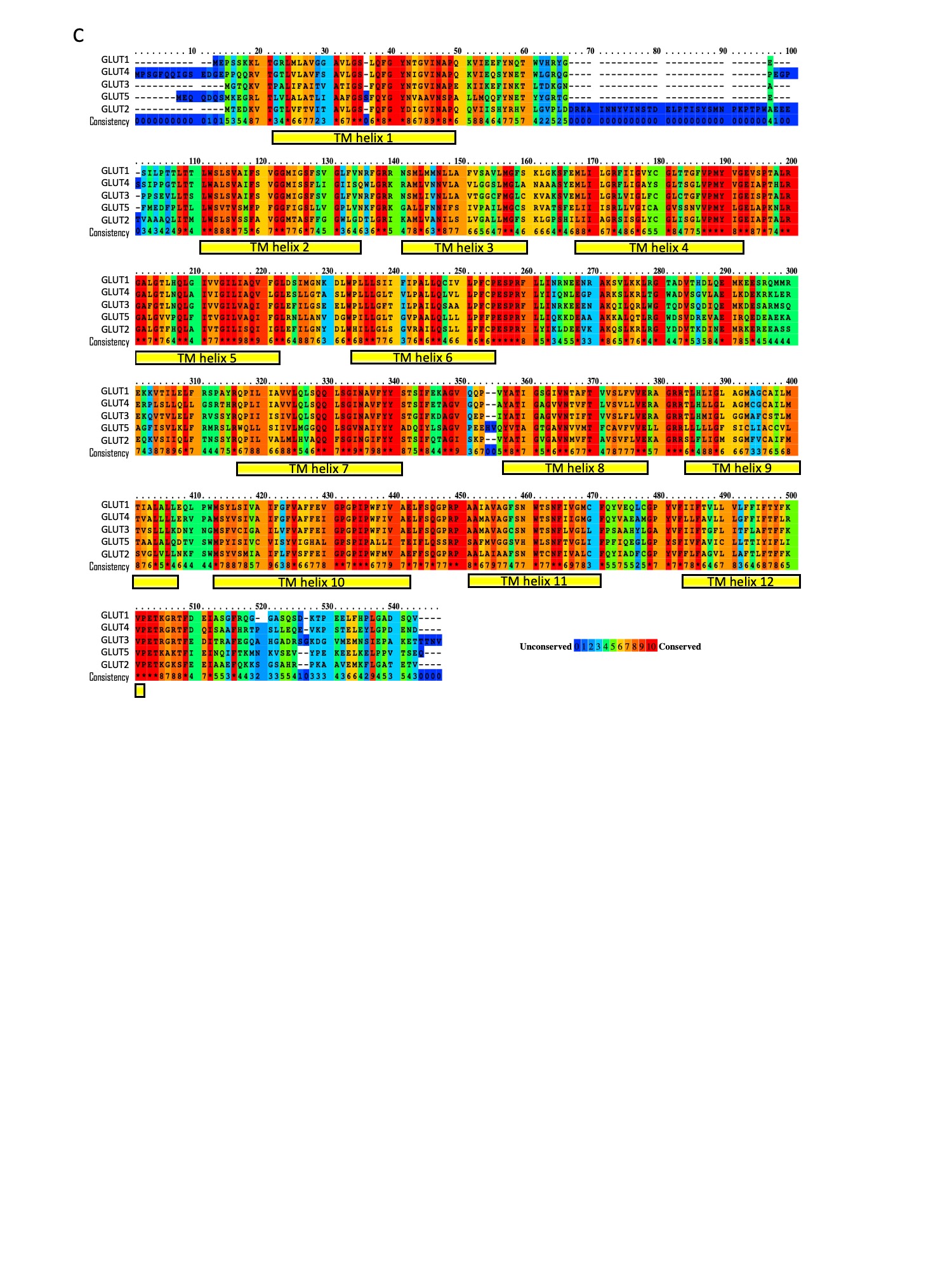


**Figure S1. GLUT homology for GLUT1-5.** (A, B) The homology models were generated in MOE (https://www.chemcomp.com) based on the crystal structure of GLUT1 (PDB ID: 4PYP), with the sequence alignment color-coded; blue regions show sequence conservation, red ones show the highest sequence variation. GLUT2 is more closely related to GLUT1, 3, and 4 (52-65% sequence identity) as compared with GLUT5 (40% sequence identity); however, the loop between the two halves of the transporter (highlighted by a red dotted ellipse) shows a higher variation (the sequence identity among GLUT1, 2, 3, and 4 is 33-61%, and that between GLUT2 and 5 is 35%). The yellow ellipse labeled G2i shows the approximate location of GLUT2 inhibitors. (A) Homology model showing the sequence conservation (blue) for GLUT1, 2, 3, and 4. (B) Homology model showing the sequence conservation (blue) between GLUT2 and 5. (C) Amino acid sequence alignment (https://www.ibi.vu.nl/programs/pralinewww/) of GLUT1-5 shows three unconserved areas: 1) between TM helices 1 and 2 in which GLUT2 has extra ~35 amino acid residues compared with the other GLUTs; 2) between TM helices 6 and 7 (the large cytoplasmic loop highlighted by the red ellipse in A-B); and 3) after TM helix 12.

**
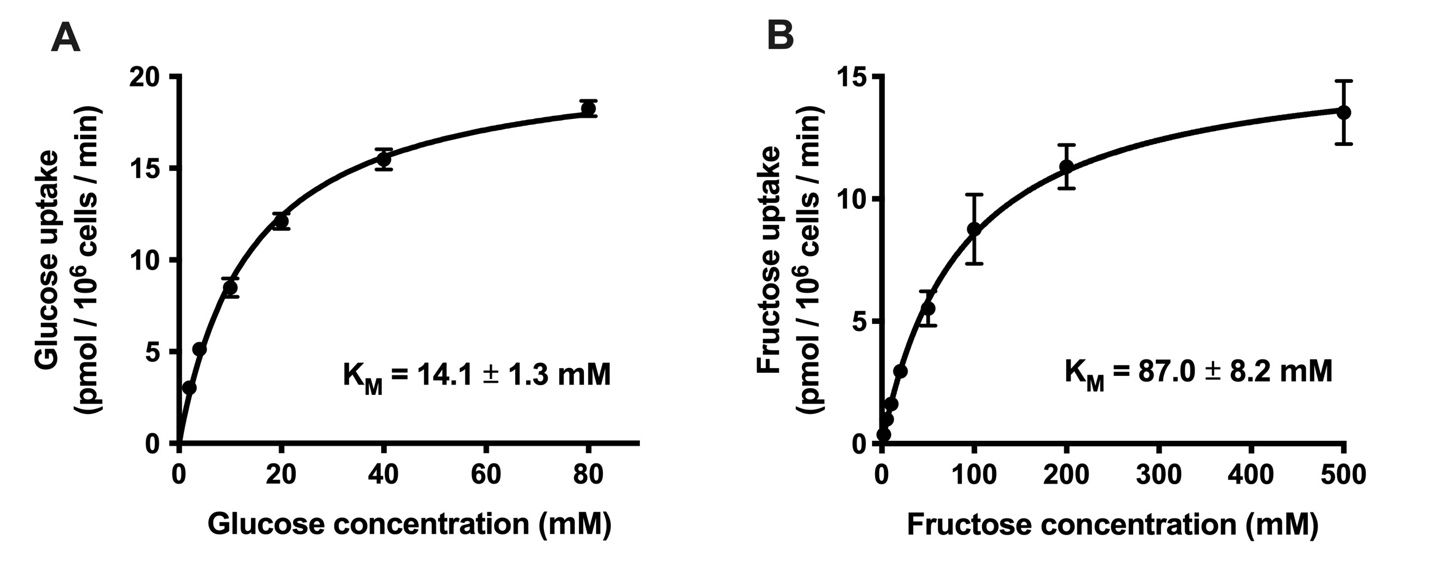
**

**Figure S2. Michaelis-Menten curves for glucose and fructose transport in GLUT2-expressing *hxt*^0^ yeast system.** Glucose (A) and fructose (B) transport activity by GLUT2_∆loopS_Q455R_ in EBY.S7 yeast cells. Transport activity was initiated by the addition of C^14^-hexose (glucose or fructose) to cells in the PBS buffer. After 10 mins, transport activity was stopped, and the radioactivity accumulated in cells was measured. See Materials and Methods for details. Error bars represent standard deviation from three independent measurements. Data analysis for calculating K_M_ values and the graphs were generated with GraphPad Prism (https://www.graphpad.com).

**Figure S3. Dixon plots for GLUT2 glucose transport inhibition by G2iA (left) and G2iB (right).** Glucose transport activity by GLUT2_∆loopS_Q455R_ in EBY.S7 yeast cells was determined at 7, 15, and 30 mM glucose, in the absence or presence of different inhibitor concentrations. Transport activity was initiated by the addition of C^14^- glucose to cells in the PBS buffer. After 10 mins, transport activity was stopped, and the radioactivity accumulated in cells was measured. See Materials and Methods for details. Data analysis for calculating K_i_ values and the graphs were generated with GraphPad Prism (<https://www.graphpad.com>). Error bars represent standard deviation from three measurements.

A. G2iA


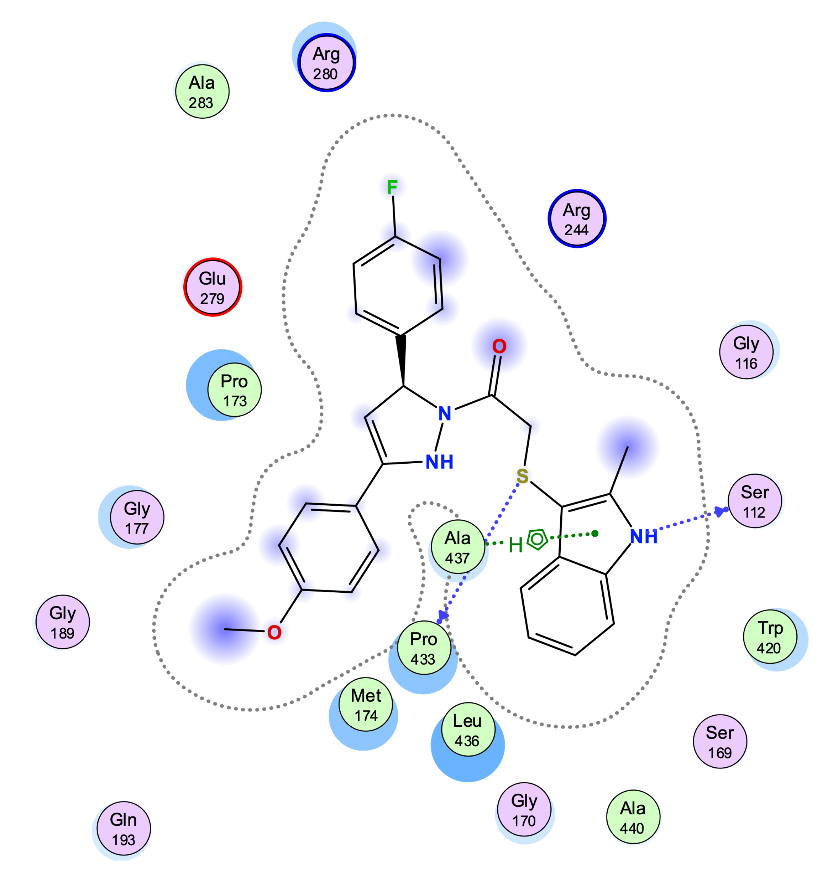


B. G2iB


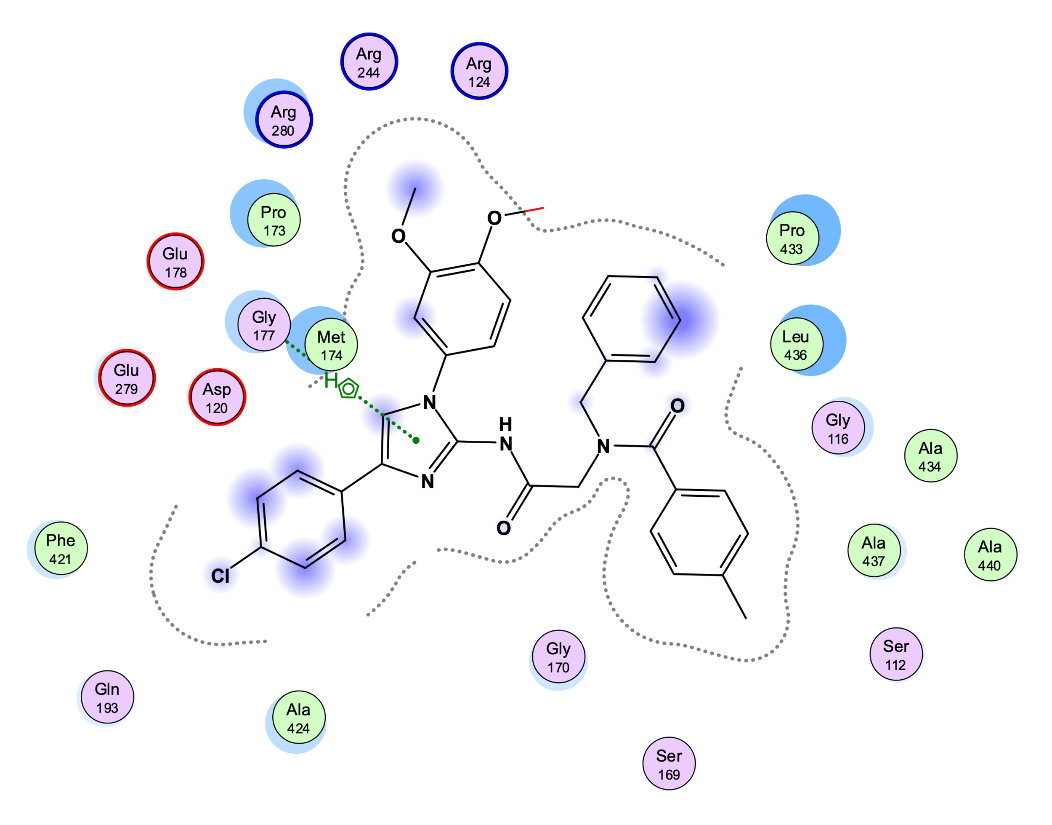


C. G2iC D. G2iD


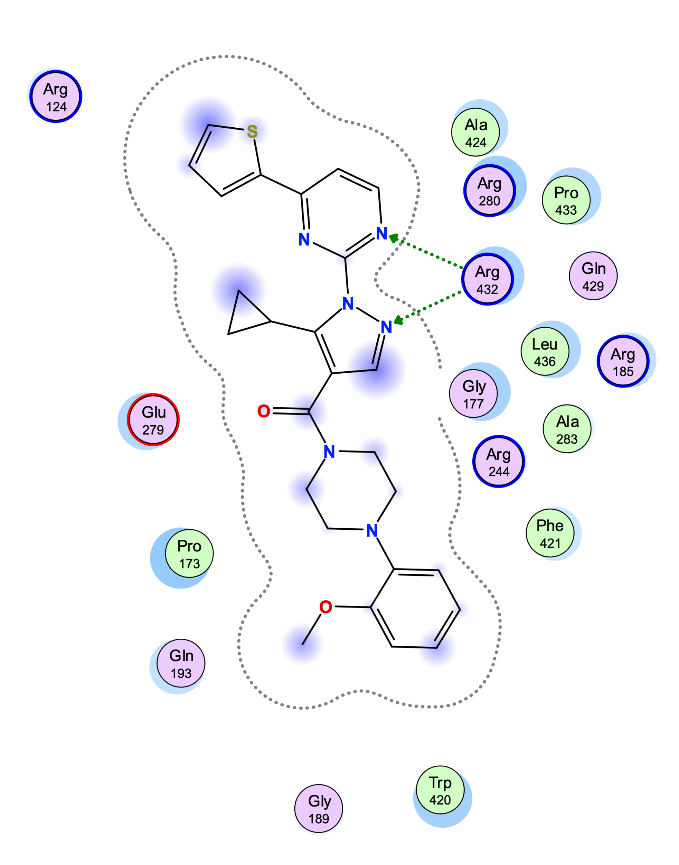

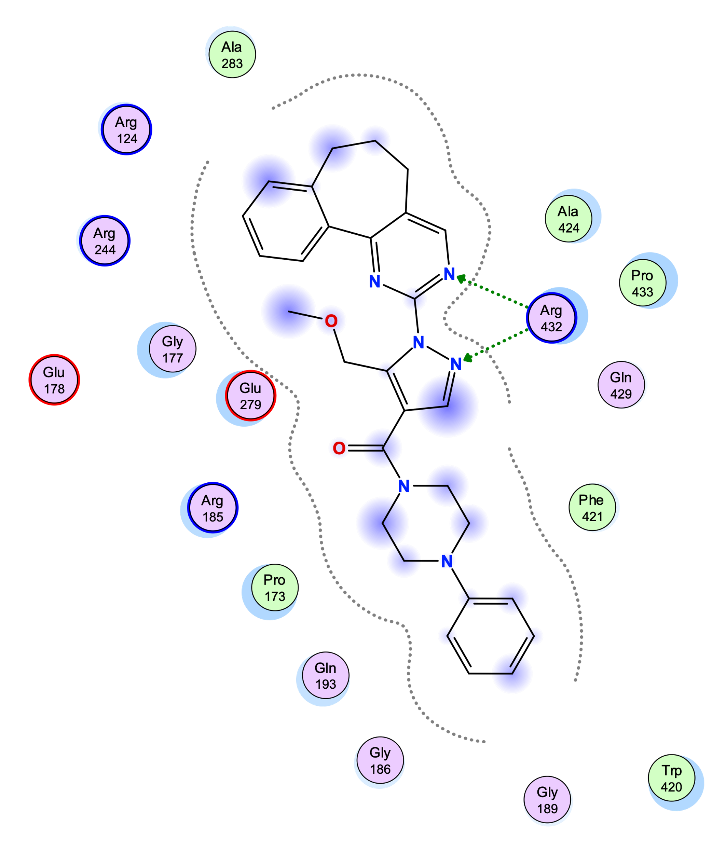


E. G2iE F. G2iF


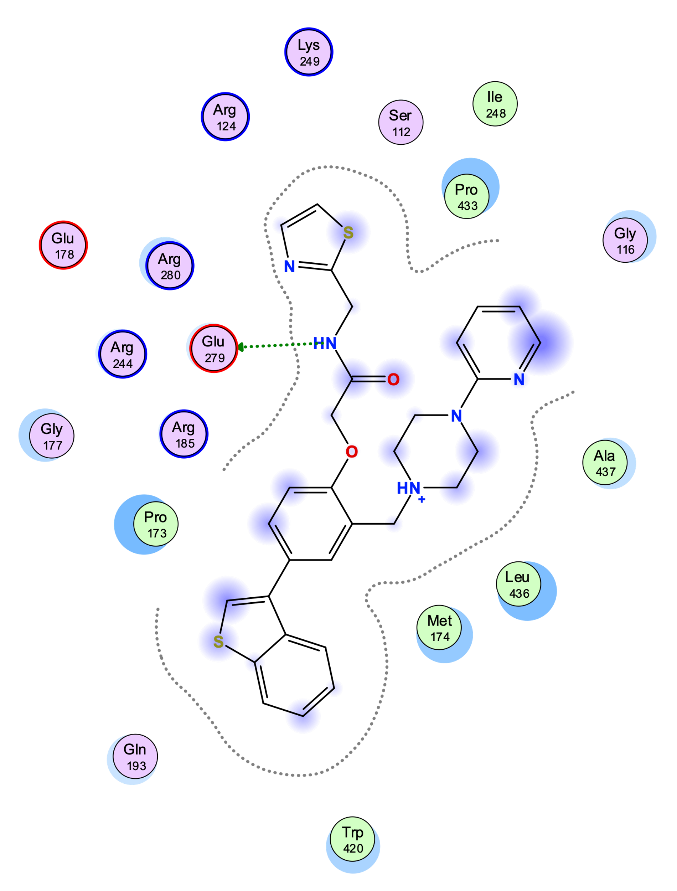

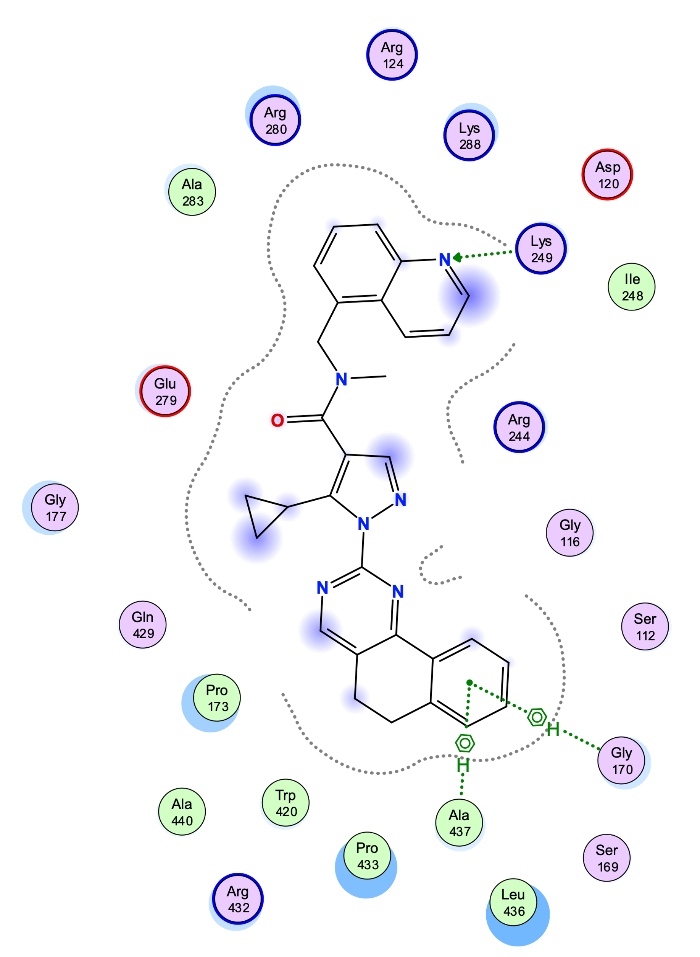


G. G2iG H. G2iF


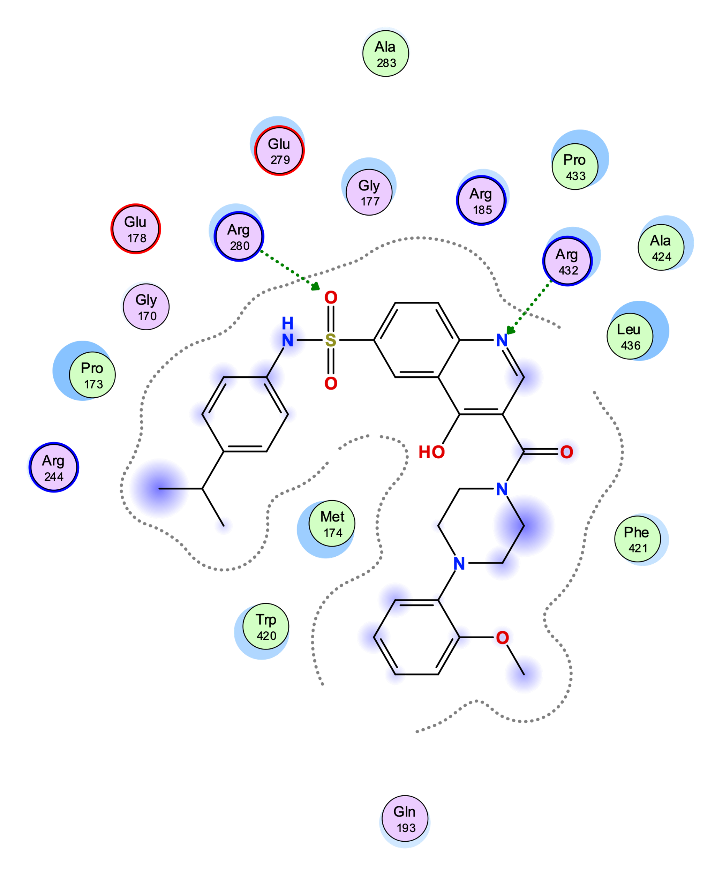

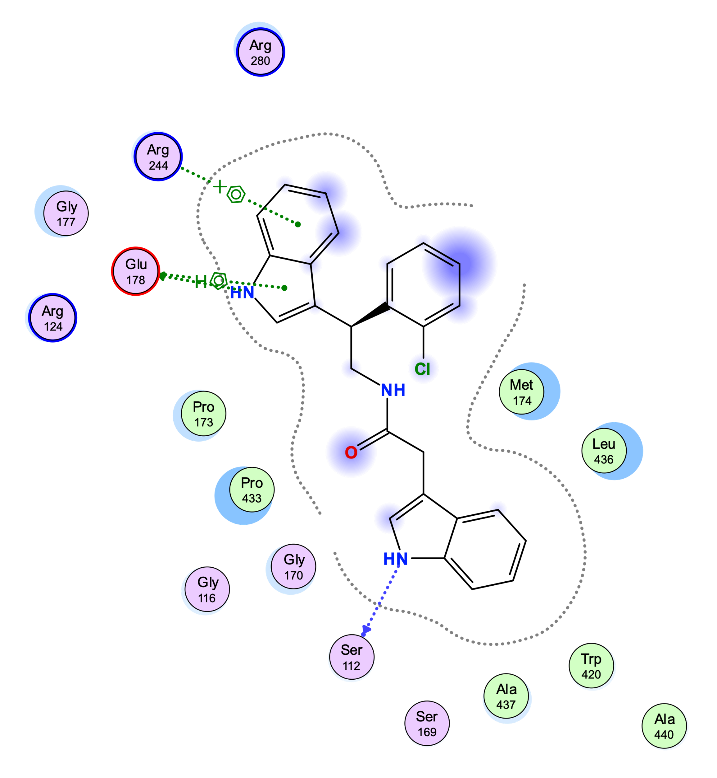


I. G2iI J. G2iJ


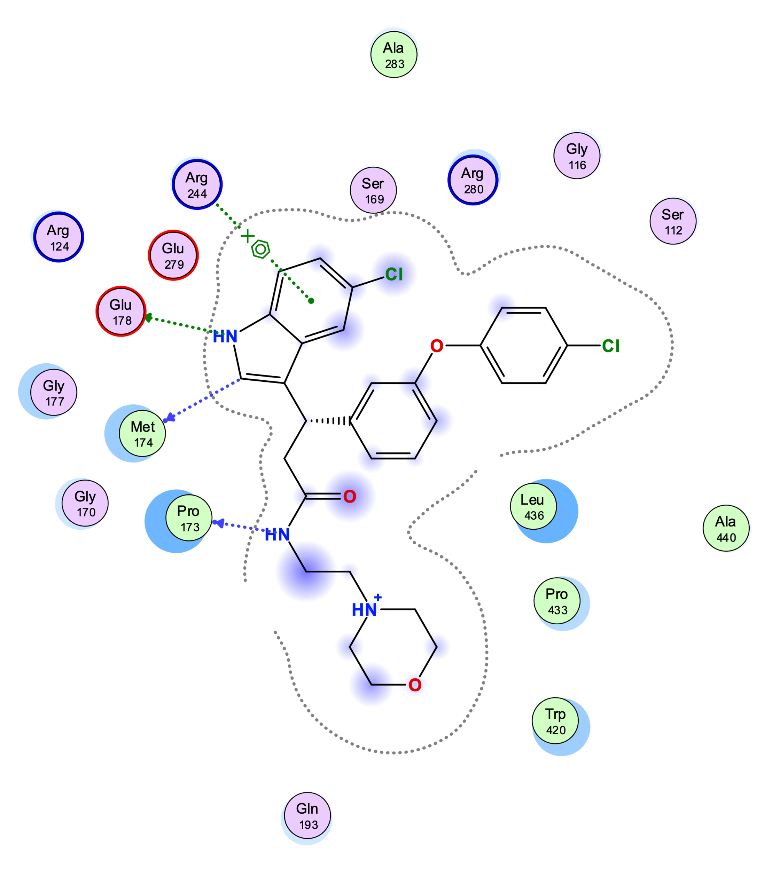

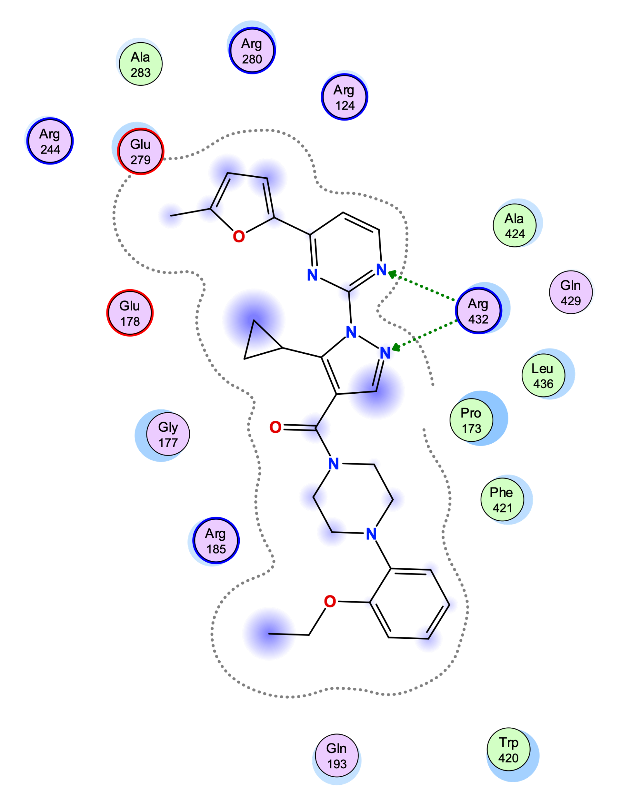


K. G2iK


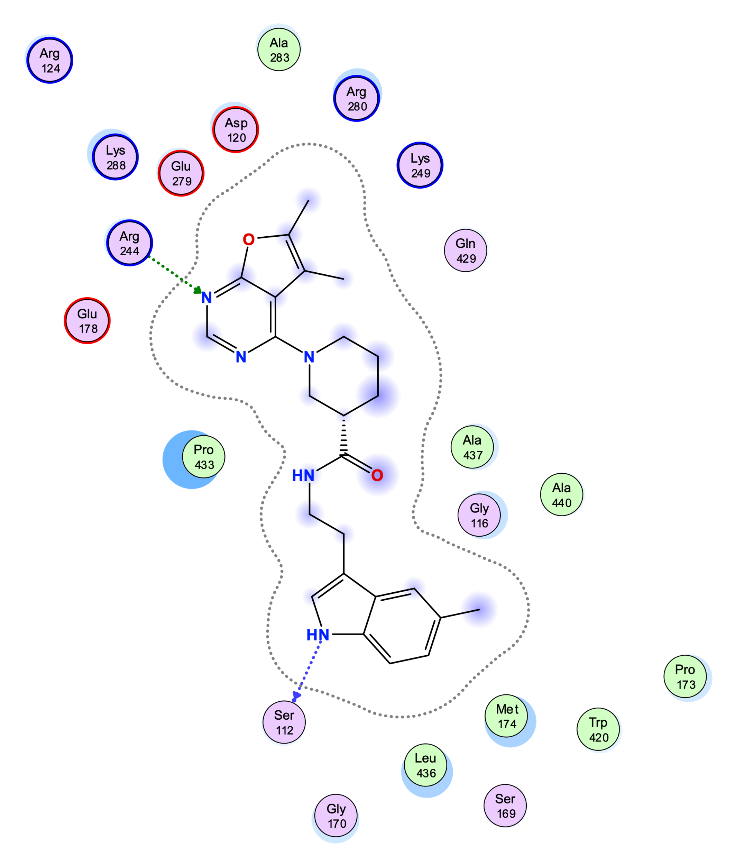


L. Legend


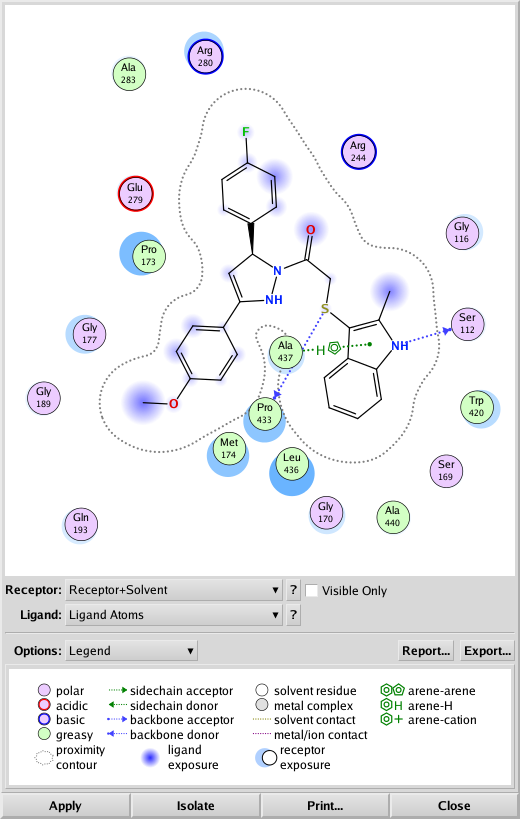


**Figure S4. Protein-ligand interactions for docked GLUT2 inhibitors to the inward-facing conformation GLUT2 model.** The ligand-protein interaction diagrams for G2iA (A), G2iB (B), G2iC (C), G2iD (D), G2iE (E), G2iF (F), G2iG (G), G2iH (H), G2iI (I), G2iJ (J), and G2iK (K), were generated in MOE ([www.chemcomp.com](http://www.chemcomp.com)) with the program “Ligand Interactions”. (L) Legend for the type of residues and ligand-protein interactions in (A-K).

**Table S1. *In silico* ligand screening candidates tested for GLUT2 transport inhibition.** The structure identifier (STRUCTURE_ID), commercial information (SUPPLIER and CATALOG #), chemical name (CHEM_NAME), SMILES code (SMILES), and the molecular formula (MF) are indicated for each compound (except for some unavailable chemicals’ names). Identified potent GLUT2 inhibitors (G2iA-K) are in bold, red font.

| STRUCTURE_ID | SUPPLIER | SUPPLIER_CATALOG # | CHEM_NAME | SMILES | MF |
| --- | --- | --- | --- | --- | --- |
| 448434472 | Ark Pharm | AK242035 |  | N1(CCN(CC1)c2ncc(cc2)c3cc4[n](cc(c4c(c3)C(=O)NCc5c(nc(cc5C)C)O)C)C(C)C)C | C31H38O2N6 |
| 403941836 | Ark Pharm | AK317596 | 5-(ethyl(tetrahydro-2H-pyran-4-yl)amino)-N-((2-hydroxy-4,6-dimethylpyridin-3-yl)methyl)-4-methyl-4'-(morpholinomethyl)-[1,1'-biphenyl]-3-carboxamide | N5(CCOCC5)Cc1ccc(cc1)c2cc(c(c(c2)C(=O)NCc4c(nc(cc4C)C)O)C)N(C3CCOCC3)CC | C34H44O4N4 |
| 407639426 | Target Molecule Corp. | T1905 | 1-cyclopentyl-N-((2-hydroxy-4,6-dimethylpyridin-3-yl)methyl)-6-(4-(morpholinomethyl)phenyl)-1H-indazole-4-carboxamide | N6(CCOCC6)Cc1ccc(cc1)c2cc3[n](ncc3c(c2)C(=O)NCc5c(nc(cc5C)C)O)C4CCCC4 | C32H37O3N5 |
| 27829088 | ChemBridge Corporation | 5340763 | 2-(3,5-diphenyl-4,5-dihydro-1H-pyrazol-1-yl)-4-(4-fluorophenyl)-6-phenylpyrimidine | Fc1ccc(cc1)c2nc(nc(c2)c6ccccc6)N3NC(=CC3c5ccccc5)c4ccccc4 | C31H23N4F1 |
| 30023243 | ChemBridge Corporation | 5720478 | 4-(4-benzhydryl-1-piperazinyl)-1-phenyl-6-(1-piperidinyl)-1H-pyrazolo[3,4-d]pyrimidine | N3(CCN(CC3)c4nc(nc6[n](ncc64)c7ccccc7)N5CCCCC5)C(c2ccccc2)c1ccccc1 | C33H35N7 |
| 466119877 | **ChemBridge Corporation** | **77390155** | **2-{5-(methoxymethyl)-4-[(4-phenyl-1-piperazinyl)carbonyl]-1H-pyrazol-1-yl}-6,7-dihydro-5H-benzo[6,7]cyclohepta[1,2-d]pyrimidine (G2iD)** | **[n]4(ncc(c4COC)C(=O)N5CCN(CC5)c6ccccc6)c1nc2c(cn1)CCCc3c2cccc3** | **C29H30O2N6** |
| 549939793 | ChemBridge Corporation | 94887258 |  | FC(F)(F)c1cc(ccc1)OC[C@@H]2CN(C[C@@H](C2)C(=O)NCCc4c5c([nH]c4)cccc5)CCc3ccccc3 | C32H34O2N3F3 |
| 549930126 | ChemBridge Corporation | 26385775 |  | N2(C[C@H](C[C@H](C2)C(=O)NCCc5c6c([nH]c5)cccc6)COc3cc4c(cc3)CCC4)Cc1cc(c(cc1)OC)OC | C35H41O4N3 |
| 347006762 | ChemBridge Corporation | 40266327 | (3R,5S)-5-[(2,3-dihydro-1H-inden-5-yloxy)methyl]-1-(2-naphthylmethyl)-N-(2-phenylethyl)-3-piperidinecarboxamide | N3(C[C@H](C[C@H](C3)C(=O)NCCc6ccccc6)COc4cc5c(cc4)CCC5)Cc1cc2c(cc1)cccc2 | C35H38O2N2 |
| 181925102 | ChemBridge Corporation | 18817511 | 3-(1-benzothien-7-yl)-1-(cyclopropylmethyl)-6-[(4,6-dimethyl-2-oxo-1,2-dihydro-3-pyridinyl)carbonyl]-5,6,7,8-tetrahydro[1,6]naphthyridin-2(1H)-one | [s]1c2c(cc1)cccc2C3=CC4=C(N(C3=O)CC6CC6)CCN(C4)C(=O)c5c(nc(cc5C)C)O | C28H27O3N3S1 |
| 181950723 | ChemBridge Corporation | 42621764 | N-benzyl-N,1-dimethyl-5-(spiro[indene-1,4'-piperidin]-1'-yl)-4,5,6,7-tetrahydro-1H-indazole-3-carboxamide | [n]1(nc(c3c1CCC(C3)N4CCC5(CC4)c6c(cccc6)C=C5)C(=O)N(Cc2ccccc2)C)C | C30H34O1N4 |
| 181921786 | ChemBridge Corporation | 16753878 | 1,8-bis(2-phenylethyl)-3-(3-pyridinylmethyl)-1,3,8-triazaspiro[4.5]decane-2,4-dione | N2(C4(CCN(CC4)CCc5ccccc5)C(=O)N(C2=O)Cc3cnccc3)CCc1ccccc1 | C29H32O2N4 |
| 181940757 | ChemBridge Corporation | 32425739 | 8-(2,6-dimethyl-5-heptenyl)-1-(2-phenylethyl)-3-(3-pyridinylmethyl)-1,3,8-triazaspiro[4.5]decane-2,4-dione | N2(C4(CCN(CC4)CC(CCC=C(C)C)C)C(=O)N(C2=O)Cc3cnccc3)CCc1ccccc1 | C30H40O2N4 |
| 181915466 | ChemBridge Corporation | 12052429 | 1-(4-methoxybenzyl)-4-{[(1R,2R)-2-phenylcyclopropyl]carbonyl}-6-(3-pyridinylmethoxy)-1,4-diazepan-2-one | N3(CC(CN(C(=O)C3)Cc5ccc(cc5)OC)OCc4cnccc4)C(=O)[C@H]1[C@@H](C1)c2ccccc2 | C29H31O4N3 |
| 182070366 | ChemBridge Corporation | 94419756 | 8-(1H-indol-3-ylmethyl)-3-isobutyl-1-[2-(4-methoxyphenyl)ethyl]-1,3,8-triazaspiro[4.5]decane-2,4-dione | [nH]1c2c(c(c1)CN3CCC4(N(C(=O)N(C4=O)CC(C)C)CCc5ccc(cc5)OC)CC3)cccc2 | C29H36O3N4 |
| 181349814 | **ChemBridge Corporation** | **57791277** | **2-(4-(1-benzothien-3-yl)-2-{[4-(2-pyridinyl)-1-piperazinyl]methyl}phenoxy)-N-(1,3-thiazol-2-ylmethyl)acetamide (G2iE)** | **[s]1c(ncc1)CNC(=O)COc2c(cc(cc2)c5c6c([s]c5)cccc6)CN3CCN(CC3)c4ncccc4** | **C30H29O2N5S2** |
| 466125339 | ChemBridge Corporation | 87775506 |  | [s]1c(c(cc1C)c2nc(ncc2)[n]3ncc(c3C5CC5)C(=O)N(Cc4cc(ccc4)OC)C)C | C26H27O2N5S1 |
| 181305778 | **ChemBridge Corporation** | **26258872** | **2-(5-cyclopropyl-4-{[4-(2-ethoxyphenyl)-1-piperazinyl]carbonyl}-1H-pyrazol-1-yl)-4-(5-methyl-2-furyl)pyrimidine (G2iJ)** | **[n]3(ncc(c3C6CC6)C(=O)N4CCN(CC4)c5c(cccc5)OCC)c1nc(ccn1)c2[o]c(cc2)C** | **C28H30O3N6** |
| 181336555 | ChemBridge Corporation | 47200727 | methyl 3-[4-{1-[(2-methyl-1H-indol-3-yl)methyl]-4-piperidinyl}-2-(4-pyridinyl)-5-pyrimidinyl]phenyl ether | N3(CCC(CC3)c4nc(ncc4c6cc(ccc6)OC)c5ccncc5)Cc1c2c([nH]c1C)cccc2 | C31H31O1N5 |
| 181267787 | ChemBridge Corporation | 11197728 | 2-[2,5-dioxo-3-phenyl-1-(3-pyridinylmethyl)-3-pyrrolidinyl]-N-methyl-N-[2-(trifluoromethyl)benzyl]acetamide | FC(F)(F)c1c(cccc1)CN(C)C(=O)CC3(CC(=O)N(C3=O)Cc4cnccc4)c2ccccc2 | C27H24O3N3F3 |
| 181971369 | ChemBridge Corporation | 68833669 | {4-{[4-(2,5-dimethylphenyl)-1-piperazinyl]carbonyl}-1-[4-(2-thienyl)-2-pyrimidinyl]-1H-pyrazol-5-yl}methyl methyl ether | [s]1c(ccc1)c2nc(ncc2)[n]3ncc(c3COC)C(=O)N4CCN(CC4)c5c(ccc(c5)C)C | C26H28O2N6S1 |
| 181931148 | ChemBridge Corporation | 23777777 | 3-({3-[4-(2-methoxyphenyl)-1-piperazinyl]-1-piperidinyl}carbonyl)-4,6-dimethyl-2(1H)-pyridinone | N3(CCN(CC3)c4c(cccc4)OC)C1CN(CCC1)C(=O)c2c(nc(cc2C)C)O | C24H32O3N4 |
| 181278705 | **ChemBridge Corporation** | **15094108** | **2-(5-cyclopropyl-4-{[4-(2-methoxyphenyl)-1-piperazinyl]carbonyl}-1H-pyrazol-1-yl)-4-(2-thienyl)pyrimidine (G2iC)** | **[s]1c(ccc1)c2nc(ncc2)[n]3ncc(c3C6CC6)C(=O)N4CCN(CC4)c5c(cccc5)OC** | **C26H26O2N6S1** |
| 181284072 | ChemBridge Corporation | 16803568 | 6-[(4'-methyl[1,1'-biphenyl]-4-yl)carbonyl]-1-{[4-(2-pyridinyl)-1-piperazinyl]carbonyl}-6-azaspiro[2.5]octane | N2(CCN(CC2)C(=O)C3C4(CCN(CC4)C(=O)c5ccc(cc5)c6ccc(cc6)C)C3)c1ncccc1 | C31H34O2N4 |
| 181268085 | ChemBridge Corporation | 11381046 | 2-{5-cyclopropyl-4-[(4-phenyl-1-piperazinyl)carbonyl]-1H-pyrazol-1-yl}-4-(3-methoxyphenyl)pyrimidine | [n]3(ncc(c3C6CC6)C(=O)N4CCN(CC4)c5ccccc5)c1nc(ccn1)c2cc(ccc2)OC | C28H28O2N6 |
| 181962616 | ChemBridge Corporation | 56596371 | 2-[4-[(4-cyclopentyl-1-piperazinyl)carbonyl]-5-(methoxymethyl)-1H-pyrazol-1-yl]-4-(2,5-dimethyl-3-thienyl)pyrimidine | [s]1c(c(cc1C)c2nc(ncc2)[n]3ncc(c3COC)C(=O)N4CCN(CC4)C5CCCC5)C | C25H32O2N6S1 |
| 182001206 | **ChemBridge Corporation** | **88168217** | **5-cyclopropyl-1-(5,6-dihydrobenzo[h]quinazolin-2-yl)-N-methyl-N-(5-quinolinylmethyl)-1H-pyrazole-4-carboxamide (G2iF)** | **[n]4(ncc(c4C7CC7)C(=O)N(Cc5c6c(nccc6)ccc5)C)c1nc2c(cn1)CCc3c2cccc3** | **C30H26O1N6** |
| 181149526 | ChemBridge Corporation | 35941485 | N-{[3-(1-benzofuran-2-yl)-1-benzyl-1H-pyrazol-4-yl]methyl}-2-(3,5-dimethyl-1H-pyrazol-4-yl)-N-methylethanamine | [n]2(nc(c(c2)CN(CCc5c([nH]nc5C)C)C)c3[o]c4c(c3)cccc4)Cc1ccccc1 | C27H29O1N5 |
| 181121814 | ChemBridge Corporation | 24355885 | 3-[(3-{3-oxo-3-[4-(2-pyridinyl)-1-piperazinyl]propyl}-1-piperidinyl)carbonyl]-1H-indazole | N2(CCN(CC2)C(=O)CCC3CN(CCC3)C(=O)c4n[nH]c5c4cccc5)c1ncccc1 | C25H30O2N6 |
| 947315724 | ChemBridge Corporation | 78029423 |  | [s]1c2c(c(c1)C(=O)N3CCNC(=O)[C@@H](NC(=O)[C@@H](N(C(=O)[C@@H](NC(=O)C3)Cc5c6c([nH]c5)cccc6)C)C)Cc4ccccc4)CCCC2 | C37H42O5N6S1 |
| 258707156 | Princeton BioMolecular Research, Inc. | OSSL_325802 | 5-(4-methylphenyl)-2-phenyl-7-{4-[2-(4-pyridinyl)ethyl]-1,4-diazepan-1-yl}[1,3]oxazolo[4,5-d]pyrimidine | N2(CCN(CCC2)c3nc(nc5nc([o]c53)c6ccccc6)c4ccc(cc4)C)CCc1ccncc1 | C30H30O1N6 |
| 34977075 | Princeton BioMolecular Research, Inc. | OSSL_392390 | 7-[3-(1,3-benzothiazol-2-ylsulfanyl)propyl]-3-methyl-8-(4-phenyl-1-piperazinyl)-3,7-dihydro-1H-purine-2,6-dione | [s]1c2c(nc1SCCC[n]3c4c(nc3N5CCN(CC5)c6ccccc6)N(C(=O)NC4=O)C)cccc2 | C26H27O2N7S2 |
| 35943973 | Princeton BioMolecular Research, Inc. | OSSL_575565 | 2-(3-[1,1'-biphenyl]-4-yl-1-{[5-(5-chloro-2-thienyl)-7-(trifluoromethyl)pyrazolo[1,5-a]pyrimidin-2-yl]carbonyl}-4,5-dihydro-1H-pyrazol-5-yl)phenol | FC(F)(F)C1=CC(=Nc3[n]1nc(c3)C(=O)N4NC(=CC4c7c(cccc7)O)c5ccc(cc5)c6ccccc6)c2[s]c(cc2)Cl | C33H21O2N5Cl1S1F3 |
| 63176616 | Princeton BioMolecular Research, Inc. | OSSL_586205 | 2-[3,5-bis(2,4-dimethoxyphenyl)-1H-pyrazol-1-yl]-4-(4-fluorophenyl)-6-(trifluoromethyl)pyrimidine | FC(F)(F)c1nc(nc(c1)c5ccc(cc5)F)[n]2nc(cc2c4c(cc(cc4)OC)OC)c3c(cc(cc3)OC)OC | C30H24O4N4F4 |
| 63178386 | Princeton BioMolecular Research, Inc. | OSSL_587015 | 2-[3,5-bis(3,4-dimethoxyphenyl)-1H-pyrazol-1-yl]-4-(4-fluorophenyl)-6-(trifluoromethyl)pyrimidine | FC(F)(F)c1nc(nc(c1)c5ccc(cc5)F)[n]2nc(cc2c4cc(c(cc4)OC)OC)c3cc(c(cc3)OC)OC | C30H24O4N4F4 |
| 63178397 | Princeton BioMolecular Research, Inc. | OSSL_589046 | 2-{3,5-bis[4-(difluoromethoxy)phenyl]-1H-pyrazol-1-yl}-4-phenyl-6-(trifluoromethyl)pyrimidine | FC(F)(F)c1nc(nc(c1)c5ccccc5)[n]2nc(cc2c4ccc(cc4)OC(F)F)c3ccc(cc3)OC(F)F | C28H17O2N4F7 |
| 40904376 | Princeton BioMolecular Research, Inc. | OSSL_768205 | N-benzyl-1-(1H-indol-3-ylmethyl)-N-(2-phenylethyl)-4-piperidinamine oxalate | N3(CCC(CC3)N(CCc5ccccc5)Cc4ccccc4)Cc1c2c([nH]c1)cccc2.OC(=O)C(=O)O | C31H35O4N3 |
| 35085263 | Scientific Exchange, Inc. | M-033644 | 6-amino-3-(1-naphthyl)-4-[4-(1-naphthylmethoxy)phenyl]-1,4-dihydropyrano[2,3-c]pyrazole-5-carbonitrile | [nH]1nc(c4c1OC(=C(C4c5ccc(cc5)OCc6c7c(ccc6)cccc7)C#N)N)c2c3c(ccc2)cccc3 | C34H24O2N4 |
| 27659180 | Specs | AG-205/11132108 | 2-(3-{2-[3-(4-fluorophenyl)-5-(4-isopropylphenyl)-2,5-dihydro-1H-pyrazol-1-yl]-1,3-thiazol-4-yl}phenyl)-1H-isoindole-1,3(2H)-dione | Fc1ccc(cc1)C2=CC(N(N2)c4[s]cc(n4)c5cc(ccc5)N6C(=O)c7c(cccc7)C6=O)c3ccc(cc3)C(C)C | C35H27O2N4S1F1 |
| 27916761 | Specs | AG-690/13508135 | 4-(4-tert-butylphenyl)-2-{[2-oxo-2-(10H-phenothiazin-10-yl)ethyl]sulfanyl}-6-phenylnicotinonitrile | S1c2c(cccc2)N(c6c1cccc6)C(=O)CSc3nc(cc(c3C#N)c5ccc(cc5)C(C)(C)C)c4ccccc4 | C36H29O1N3S2 |
| 27824288 | Specs | AG-690/40750941 | N-(4-chlorobenzyl)-N-(1-naphthyl)-2-[(2-phenyl-4-quinazolinyl)sulfanyl]acetamide | S(CC(=O)N(Cc6ccc(cc6)Cl)c4c5c(ccc4)cccc5)c1nc(nc3c1cccc3)c2ccccc2 | C33H24O1N3Cl1S1 |
| 27883605 | Specs | AG-690/40753767 | 2-phenoxyethyl 4-[2-(benzyloxy)phenyl]-7-(3,4-dimethoxyphenyl)-2-methyl-5-oxo-1,4,5,6,7,8-hexahydro-3-quinolinecarboxylate | N1C2=C(C(C(=C1C)C(=O)OCCOc6ccccc6)c4c(cccc4)OCc5ccccc5)C(=O)CC(C2)c3cc(c(cc3)OC)OC | C40H39O7N1 |
| 27659184 | Specs | AG-205/11132170 | N-(4-{2-[3-(4-fluorophenyl)-5-(4-isopropylphenyl)-2,5-dihydro-1H-pyrazol-1-yl]-1,3-thiazol-4-yl}phenyl)-4-methylbenzenesulfonamide | Fc1ccc(cc1)C2=CC(N(N2)c4[s]cc(n4)c5ccc(cc5)N[S](=O)(=O)c6ccc(cc6)C)c3ccc(cc3)C(C)C | C34H31O2N4S2F1 |
| 27644832 | Specs | AN-919/14712055 | 4-(4-methoxyphenyl)-2-(10H-phenothiazin-10-ylcarbonyl)-6-phenylthieno[2,3-b]pyridin-3-amine | [s]1c2nc(cc(c2c(c1C(=O)N5c6c(cccc6)Sc7c5cccc7)N)c4ccc(cc4)OC)c3ccccc3 | C33H23O2N3S2 |
| 35975457 | InterBioScreen Ltd. | STOCK7S-69659 | (8S,8aR,11aS)-10-(2-naphthyl)-9,11-dioxo-N-[3-(trifluoromethyl)phenyl]-8a,9,10,11,11a,11b-hexahydro-8H-pyrrolo[3',4':3,4]pyrrolo[2,1-a]isoquinoline-8-carboxamide | FC(F)(F)c1cc(ccc1)NC(=O)[C@H]2N3C([C@@H]5[C@H]2C(=O)N(C5=O)c6cc7c(cc6)cccc7)c4c(cccc4)C=C3 | C32H22O3N3F3 |
| 29332280 | InterBioScreen Ltd. | STOCK7S-68264 | 3-benzhydryl 1-methyl (1S,2R,3R)-1-cyano-2-[4-(dimethylamino)phenyl]-1,2,3,10b-tetrahydropyrrolo[2,1-a]isoquinoline-1,3-dicarboxylate | N21[C@H]([C@@H]([C@@](C2c6c(cccc6)C=C1)(C(=O)OC)C#N)c5ccc(cc5)N(C)C)C(=O)OC(c4ccccc4)c3ccccc3 | C37H33O4N3 |
| 29351964 | InterBioScreen Ltd. | STOCK7S-65756 | benzhydryl (8S,8aR,11aS)-10-(4-methoxyphenyl)-9,11-dioxo-8a,9,10,11,11a,11b-hexahydro-8H-pyrrolo[3',4':3,4]pyrrolo[2,1-a]isoquinoline-8-carboxylate | N21[C@@H]([C@H]5[C@@H](C2c7c(cccc7)C=C1)C(=O)N(C5=O)c6ccc(cc6)OC)C(=O)OC(c4ccccc4)c3ccccc3 | C35H28O5N2 |
| 29728600 | Life Chemicals Inc. | F0417-2422 | ethyl 5-({2-[(4-benzoylbenzoyl)amino]benzoyl}oxy)-2-methyl-1-phenyl-1H-indole-3-carboxylate | [n]2(c3c(c(c2C)C(=O)OCC)cc(cc3)OC(=O)c4c(cccc4)NC(=O)c5ccc(cc5)C(=O)c6ccccc6)c1ccccc1 | C39H30O6N2 |
| 30865539 | **Life Chemicals Inc.** | **F0575-0046** | **4-(5-(4-fluorophenyl)-1-{[(2-methyl-1H-indol-3-yl)sulfanyl]acetyl}-4,5-dihydro-1H-pyrazol-3-yl)phenyl methyl ether (G2iA)** | **Fc1ccc(cc1)C2N(NC(=C2)c5ccc(cc5)OC)C(=O)CSc3c4c([nH]c3C)cccc4** | **C27H24O2N3S1F1** |
| 29791344 | Life Chemicals Inc. | F0721-0768 | N-{[5-{[2-(3,4-dihydro-1(2H)-quinolinyl)-2-oxoethyl]sulfanyl}-4-(2-methoxyphenyl)-4H-1,2,4-triazol-3-yl]methyl}-4-[(dimethylamino)sulfonyl]benzamide | [S](=O)(=O)(N(C)C)c1ccc(cc1)C(=O)NCc2[n](c(nn2)SCC(=O)N4CCCc5c4cccc5)c3c(cccc3)OC | C30H32O5N6S2 |
| 30335515 | Life Chemicals Inc. | F0913-4612 | 3-{[4-(2,5-dimethylphenyl)-1-piperazinyl]carbonyl}-6-{[4-(4-fluorophenyl)-1-piperazinyl]sulfonyl}-4(1H)-quinolinone | Fc1ccc(cc1)N2CCN(CC2)[S](=O)(=O)c3cc4c(ncc(c4O)C(=O)N5CCN(CC5)c6c(ccc(c6)C)C)cc3 | C32H34O4N5S1F1 |
| 29793191 | Life Chemicals Inc. | F0737-0110 | N-[3-(1,3-benzothiazol-2-yl)-6-methyl-4,5,6,7-tetrahydro-1-benzothien-2-yl]-4-(2,3-dihydro-1H-indol-1-ylsulfonyl)benzamide | [S](=O)(=O)(N6CCc7c6cccc7)c1ccc(cc1)C(=O)Nc2[s]c3c(c2c4[s]c5c(n4)cccc5)CCC(C3)C | C31H27O3N3S3 |
| 29606176 | Labotest | LT01321967 | N-(2-benzoyl-4-bromophenyl)-4-[(6-chloro-4-phenyl-2-quinazolinyl)amino]benzamide | Brc1cc(c(cc1)NC(=O)c3ccc(cc3)Nc4nc5c(c(n4)c6ccccc6)cc(cc5)Cl)C(=O)c2ccccc2 | C34H22O2N4Cl1Br1 |
| 65382808 | Otava | 7006549 | 4-[4-(benzyloxy)phenyl]-3-(2-hydroxyphenyl)-5-(3-pyridinylmethyl)-4,5-dihydropyrrolo[3,4-c]pyrazol-6(1H)-one | [nH]1nc(c3c1C(=O)N(C3c5ccc(cc5)OCc6ccccc6)Cc4cnccc4)c2c(cccc2)O | C30H24O3N4 |
| 77016607 | Pharmeks LTD. | PHAR335882 | 4-(bis(3-hydroxy-5-methyl-1H-pyrazol-4-yl)methyl)phenyl 4-fluorobenzoate | Fc1ccc(cc1)C(=O)Oc2ccc(cc2)C(c4c([nH]nc4O)C)c3c([nH]nc3O)C | C22H19O4N4F1 |
| 458452454 | Pharmeks LTD. | PHAR363782 |  | N3(C(=CC(=O)C(C3=O)C(CC(=O)OC)c4[o]c(cc4)c5c(cccc5)C(=O)O)C)CCc1c2c([nH]c1)cccc2 | C31H28O7N2 |
| 34979766 | Pharmeks LTD. | PHAR083314 | 8-benzoyl-4-(4-bromobenzoyl)-2-(3,5-dichlorophenyl)-3a,4,9a,9b-tetrahydro-1H-pyrrolo[3,4-a]indolizine-1,3(2H)-dione | Brc1ccc(cc1)C(=O)C2N3C(C5C2C(=O)N(C5=O)c6cc(cc(c6)Cl)Cl)C=C(C=C3)C(=O)c4ccccc4 | C30H19O4N2Cl2Br1 |
| 29363082 | Pharmeks LTD. | PHAR089794 | (11beta)-11,17-dihydroxy-3,20-dioxopregna-1,4-dien-21-yl 4-{[2-(2,2-dimethyl-4-phenyltetrahydro-2H-pyran-4-yl)ethyl]amino}-4-oxobutanoate | N(CCC6(CC(OCC6)(C)C)c5ccccc5)C(=O)CCC(=O)OCC(=O)[C@@]1([C@@]2(C(C3C([C@@]4(C(=CC(=O)C=C4)CC3)C)[C@H](C2)O)CC1)C)O | C40H53O8N1 |
| 50759467 | **Enamine** | **Z30131423** | **N-[2-(2-chlorophenyl)-2-(1H-indol-3-yl)ethyl]-2-(1H-indol-3-yl)acetamide (G2iH)** | **Clc1c(cccc1)C(CNC(=O)Cc4c5c([nH]c4)cccc5)c2c3c([nH]c2)cccc3** | **C26H22O1N3Cl1** |
| 95817411 | Enamine | Z109718458 | 5-[1,1'-biphenyl]-4-yl-N-[(4,6-dimethyl-2-oxo-1,2-dihydro-3-pyridinyl)methyl]-2-thiophenecarboxamide | [s]1c(ccc1C(=O)NCc4c(nc(cc4C)C)O)c2ccc(cc2)c3ccccc3 | C25H22O2N2S1 |
| 30206540 | Enamine | T0513-5507 | 2-({2-[2,5-dimethyl-1-(2-phenylethyl)-1H-pyrrol-3-yl]-2-oxoethyl}sulfanyl)-6-methyl-5-(4-methylphenyl)-3-phenylthieno[2,3-d]pyrimidin-4(3H)-one | [s]1c2c(c(c1C)c6ccc(cc6)C)C(=O)N(C(=N2)SCC(=O)c4c([n](c(c4)C)CCc5ccccc5)C)c3ccccc3 | C36H33O2N3S2 |
| 29399326 | Enamine | Z19304338 | N-(9-ethyl-9H-carbazol-3-yl)-2-{[5-(1H-indol-3-yl)-1,3,4-oxadiazol-2-yl]sulfanyl}acetamide | S(CC(=O)Nc4cc5c([n](c6c5cccc6)CC)cc4)c1nnc([o]1)c2c3c([nH]c2)cccc3 | C26H21O2N5S1 |
| 155920809 | Enamine | Z244415796 | 3-(1-{(2E)-3-[2-(4-fluorophenyl)-1,3-thiazol-4-yl]-2-propenoyl}-1,2,3,6-tetrahydro-4-pyridinyl)-1H-pyrrolo[2,3-b]pyridine | Fc1ccc(cc1)c2[s]cc(n2)\C=C\C(=O)N3CCC(=CC3)c4c5c([nH]c4)nccc5 | C24H19O1N4S1F1 |
| 27082796 | Enamine | T5603758 | N-(2-benzoyl-4-methylphenyl)-4-[(6-methyl-4-phenyl-2-quinazolinyl)amino]benzamide | N(c4ccc(cc4)C(=O)Nc5c(cc(cc5)C)C(=O)c6ccccc6)c1nc2c(c(n1)c3ccccc3)cc(cc2)C | C36H28O2N4 |
| 112921324 | Enamine | Z220595048 | N-(1-isoquinolinylmethyl)-4-[5-(4-methoxyphenyl)-3-phenyl-4,5-dihydro-1H-pyrazol-1-yl]-4-oxobutanamide | N3(NC(=CC3c5ccc(cc5)OC)c4ccccc4)C(=O)CCC(=O)NCc1nccc2c1cccc2 | C30H28O3N4 |
| 371582429 | Enamine | Z1446647643 | 3-[1-(3-fluorobenzoyl)-5-(4-fluorophenyl)-2,5-dihydro-1H-pyrazol-3-yl]-6-methyl-4-phenyl-2-quinolinol | Fc1cc(ccc1)C(=O)N2NC(=CC2c6ccc(cc6)F)c3c(nc4c(c3c5ccccc5)cc(cc4)C)O | C32H23O2N3F2 |
| 947324579 | Enamine | Z2858952890 |  | [s]1c(ccc1C(=O)O)CN(C5CC6(N(CC5)C(=O)OC(C)(C)C)CCCCC6)C(=O)OCC2c3c(cccc3)c4c2cccc4 | C36H42O6N2S1 |
| 113915710 | Enamine | Z234513220 | 3-{1-[(1,3-diphenyl-1H-pyrazol-5-yl)carbonyl]-1,2,3,6-tetrahydro-4-pyridinyl}-1H-pyrrolo[2,3-b]pyridine | [n]2(nc(cc2C(=O)N4CCC(=CC4)c5c6c([nH]c5)nccc6)c3ccccc3)c1ccccc1 | C28H23O1N5 |
| 388606908 | Enamine | Z1558581164 | N-(2-(1-(6-chloro-4-phenylquinazolin-2-yl)-5-(4-(dimethylamino)phenyl)-2,5-dihydro-1H-pyrazol-3-yl)phenyl)methanesulfonamide | [S](=O)(=O)(Nc1c(cccc1)C2=CC(N(N2)c4nc5c(c(n4)c6ccccc6)cc(cc5)Cl)c3ccc(cc3)N(C)C)C | C32H29O2N6Cl1S1 |
| 33510957 | Enamine | Z18699620 | 2-(1H-indol-3-yl)-2-oxoethyl 3-[(2-methyl-2,3-dihydro-1H-indol-1-yl)sulfonyl]benzoate | [S](=O)(=O)(N4C(Cc5c4cccc5)C)c1cc(ccc1)C(=O)OCC(=O)c2c3c([nH]c2)cccc3 | C26H22O5N2S1 |
| 118879125 | Enamine | Z243366928 | 2-[hexyl(4-phenyl-1,3-thiazol-2-yl)amino]-2-oxoethyl 6-(1H-1,2,4-triazol-1-yl)nicotinate | [s]1c(nc(c1)c4ccccc4)N(CCCCCC)C(=O)COC(=O)c2cnc(cc2)[n]3ncnc3 | C25H26O3N6S1 |
| 428762224 | Enamine | Z1891749033 |  | Clc1cc(ccc1)N2CCN(CC2)C(=O)c3ccc(cc3)Nc4nc5c(c(n4)c6ccc(cc6)Cl)cccc5 | C31H25O1N5Cl2 |
| 30846314 | Enamine | Z19316401 | methyl 4-{3-phenyl-1-[(4-quinazolinylsulfanyl)acetyl]-4,5-dihydro-1H-pyrazol-5-yl}phenyl ether | S(CC(=O)N3NC(=CC3c5ccc(cc5)OC)c4ccccc4)c1ncnc2c1cccc2 | C26H22O2N4S1 |
| 37807463 | Enamine | Z25492200 | 2-{[4-(3,4-dimethylphenyl)-1-phenyl-1H-imidazol-2-yl]sulfanyl}-1-(2-methyl-1H-indol-3-yl)ethanone | S(CC(=O)c4c5c([nH]c4C)cccc5)c1[n](cc(n1)c3cc(c(cc3)C)C)c2ccccc2 | C28H25O1N3S1 |
| 29606134 | Enamine | T5605033 | N-(2-benzoyl-4-bromophenyl)-3-[(6-bromo-4-phenyl-2-quinazolinyl)amino]benzamide | Brc1cc(c(cc1)NC(=O)c3cc(ccc3)Nc4nc5c(c(n4)c6ccccc6)cc(cc5)Br)C(=O)c2ccccc2 | C34H22O2N4Br2 |
| 29116573 | Enamine | T0513-8000 | 2-({2-[2,5-dimethyl-1-(2-phenylethyl)-1H-pyrrol-3-yl]-2-oxoethyl}sulfanyl)-3,6-diphenylthieno[2,3-d]pyrimidin-4(3H)-one | [s]1c2c(cc1c6ccccc6)C(=O)N(C(=N2)SCC(=O)c4c([n](c(c4)C)CCc5ccccc5)C)c3ccccc3 | C34H29O2N3S2 |
| 388606909 | Enamine | Z1558581147 | 6-chloro-2-(5-(4-ethoxyphenyl)-3-(m-tolyl)-2,5-dihydro-1H-pyrazol-1-yl)-4-phenylquinazoline | Clc1cc2c(nc(nc2c6ccccc6)N3NC(=CC3c5ccc(cc5)OCC)c4cc(ccc4)C)cc1 | C32H27O1N4Cl1 |
| 406070290 | Enamine | EN300-117175 | 4-(N-(4-cyclohexylbenzyl)-2-((2,3,4,5,6-pentafluoro-N-methylphenyl)sulfonamido)acetamido)-2-hydroxybenzoic acid | Fc1c(c(c(c(c1F)F)[S](=O)(=O)N(CC(=O)N(Cc3ccc(cc3)C4CCCCC4)c2cc(c(cc2)C(=O)O)O)C)F)F | C29H27O6N2S1F5 |
| 28678113 | Vitas-M Laboratory, Ltd. | STK269113 | 2-[1,1'-biphenyl]-4-yl-2-oxoethyl 2-[1,1'-biphenyl]-4-yl-6-methyl-4-quinolinecarboxylate | n1c2c(c(cc1c5ccc(cc5)c6ccccc6)C(=O)OCC(=O)c3ccc(cc3)c4ccccc4)cc(cc2)C | C37H27O3N1 |
| 34885276 | Vitas-M Laboratory, Ltd. | STK296037 | 1,3-dioxo-2-{3-[(3-phenoxyanilino)carbonyl]phenyl}-N-(3-phenoxyphenyl)-5-isoindolinecarboxamide | N4(C(=O)c5c(ccc(c5)C(=O)Nc6cc(ccc6)Oc7ccccc7)C4=O)c1cc(ccc1)C(=O)Nc2cc(ccc2)Oc3ccccc3 | C40H27O6N3 |
| 36012691 | Vitas-M Laboratory, Ltd. | STK279195 | 4,4'-((3-bromo-4-(naphthalen-1-ylmethoxy)phenyl)methylene)bis(3-methyl-1H-pyrazol-5-ol) | Brc1c(ccc(c1)C(c5c([nH]nc5C)O)c4c([nH]nc4C)O)OCc2c3c(ccc2)cccc3 | C26H23O3N4Br1 |
| 63176574 | Vitas-M Laboratory, Ltd. | STK313895 | 2-[3,5-bis(3,4-dimethoxyphenyl)-1H-pyrazol-1-yl]-4-(2-thienyl)-6-(trifluoromethyl)pyrimidine | FC(F)(F)c1nc(nc(c1)c5[s]ccc5)[n]2nc(cc2c4cc(c(cc4)OC)OC)c3cc(c(cc3)OC)OC | C28H23O4N4S1F3 |
| 63176613 | Vitas-M Laboratory, Ltd. | STK313915 | 2-[3,5-bis(3,4-dimethoxyphenyl)-4-ethyl-1H-pyrazol-1-yl]-4-(4-fluorophenyl)-6-(trifluoromethyl)pyrimidine | FC(F)(F)c1nc(nc(c1)c5ccc(cc5)F)[n]2nc(c(c2c4cc(c(cc4)OC)OC)CC)c3cc(c(cc3)OC)OC | C32H28O4N4F4 |
| 63176630 | Vitas-M Laboratory, Ltd. | STK313922 | 2-[3,5-bis(3,4-dimethoxyphenyl)-4-ethyl-1H-pyrazol-1-yl]-4-phenyl-6-(trifluoromethyl)pyrimidine | FC(F)(F)c1nc(nc(c1)c5ccccc5)[n]2nc(c(c2c4cc(c(cc4)OC)OC)CC)c3cc(c(cc3)OC)OC | C32H29O4N4F3 |
| 63178383 | Vitas-M Laboratory, Ltd. | STK314253 | 2-[3,5-bis(3,4-dimethoxyphenyl)-4-methyl-1H-pyrazol-1-yl]-4-(3-methoxyphenyl)-6-(trifluoromethyl)pyrimidine | FC(F)(F)c1nc(nc(c1)c5cc(ccc5)OC)[n]2nc(c(c2c4cc(c(cc4)OC)OC)C)c3cc(c(cc3)OC)OC | C32H29O5N4F3 |
| 28394210 | Vitas-M Laboratory, Ltd. | STK038146 | N-benzyl-N-(4,5-diphenyl-1,3-thiazol-2-yl)-2-[(4-hydroxy-6-phenyl-2-pyrimidinyl)sulfanyl]acetamide | [s]1c(nc(c1c6ccccc6)c5ccccc5)N(Cc4ccccc4)C(=O)CSc2nc(cc(n2)c3ccccc3)O | C34H26O2N4S2 |
| 29999180 | Vitas-M Laboratory, Ltd. | STK053875 | 2-(4-tert-butylphenoxy)-N-(2-{[(4-tert-butylphenoxy)acetyl]anilino}ethyl)-N-phenylacetamide | N(CCN(c4ccccc4)C(=O)COc3ccc(cc3)C(C)(C)C)(c2ccccc2)C(=O)COc1ccc(cc1)C(C)(C)C | C38H44O4N2 |
| 28414872 | Vitas-M Laboratory, Ltd. | STK392050 | 1-(9H-fluoren-2-yl)-2-{[5-(2-furyl)-4-phenyl-4H-1,2,4-triazol-3-yl]sulfanyl}ethanone | S(CC(=O)c4cc5c(cc4)c6c(cccc6)C5)c1[n](c(nn1)c3[o]ccc3)c2ccccc2 | C27H19O2N3S1 |
| 27806338 | Vitas-M Laboratory, Ltd. | STK043440 | N-[1-(4-methoxybenzoyl)-2-methyl-1,2,3,4-tetrahydro-4-quinolinyl]-4-pentyl-N-phenylbenzamide | N2(C(CC(c5c2cccc5)N(c4ccccc4)C(=O)c3ccc(cc3)CCCCC)C)C(=O)c1ccc(cc1)OC | C36H38O3N2 |
| 39750226 | Vitas-M Laboratory, Ltd. | STL052698 | 9a-((E)-2-{4-[3-(3-chloro-4-methoxyphenyl)-1-phenyl-1H-pyrazol-4-yl]phenyl}ethenyl)-9,9-dimethyl-9,9a-dihydro-1H-imidazo[1,2-a]indol-2(3H)-one | Clc1c(ccc(c1)c2n[n](cc2c4ccc(cc4)\C=C\C65N(CC(=O)N6)c7c(cccc7)C5(C)C)c3ccccc3)OC | C36H31O2N4Cl1 |
| 158573473 | Vitas-M Laboratory, Ltd. | STK636849 | N-{2-[5-(benzyloxy)-1H-indol-3-yl]ethyl}-3-(4-pyridinyl)-1,2,4-oxadiazole-5-carboxamide | [nH]1c2c(c(c1)CCNC(=O)c4nc(n[o]4)c5ccncc5)cc(cc2)OCc3ccccc3 | C25H21O3N5 |
| 31278512 | Vitas-M Laboratory, Ltd. | STK681999 | 2-{[4-(1-adamantyl)-1-piperazinyl]carbonyl}-5,7-diphenylpyrazolo[1,5-a]pyrimidine | [n]21nc(cc2N=C(C=C1c8ccccc8)c7ccccc7)C(=O)N3CCN(CC3)C54CC6CC(C5)CC(C4)C6 | C33H35O1N5 |
| 47635094 | Vitas-M Laboratory, Ltd. | STK686647 | 4-(4-chlorophenyl)-1-(4-isopropylphenyl)-N-(3-methoxyphenyl)-5,6,7,8-tetrahydro-2a,4a-diazacyclopenta[cd]azulene-2-carboxamide | Clc1ccc(cc1)C2=C[n]3c4c(c(c3C(=O)Nc6cc(ccc6)OC)c5ccc(cc5)C(C)C)CCCCN42 | C33H32O2N3Cl1 |
| 47635071 | Vitas-M Laboratory, Ltd. | STK782131 | 4-(3,4-dimethylphenyl)-N-(4-methoxyphenyl)-1-(4-methylphenyl)-5,6,7,8-tetrahydro-2a,4a-diazacyclopenta[cd]azulene-2-carboxamide | [n]21c3c(c(c2C(=O)Nc6ccc(cc6)OC)c5ccc(cc5)C)CCCCN3C(=C1)c4cc(c(cc4)C)C | C33H33O2N3 |
| 27366974 | Vitas-M Laboratory, Ltd. | STK528173 | ethyl 3-{[(1-[(1,3-dioxo-1,3-dihydro-2H-isoindol-2-yl)methyl]-3,4-dihydro-2(1H)-isoquinolinyl)acetyl]amino}-5-methoxy-1H-indole-2-carboxylate | [nH]1c2c(c(c1C(=O)OCC)NC(=O)CN3CCc4c(cccc4)C3CN5C(=O)c6c(cccc6)C5=O)cc(cc2)OC | C32H30O6N4 |
| 27546167 | Vitas-M Laboratory, Ltd. | STK717513 | 4-ethyl-2-[4-(4-methyl-1,3-thiazol-2-yl)-1H-pyrazol-3-yl]-5-(1-naphthylmethoxy)phenol | [s]1c(nc(c1)C)c2c[nH]nc2c3c(cc(c(c3)CC)OCc4c5c(ccc4)cccc5)O | C26H23O2N3S1 |
| 27304178 | Vitas-M Laboratory, Ltd. | STK526378 | ethyl 6-amino-2-({[4-(4-chlorophenyl)-3-cyano-6-(2-thienyl)-2-pyridinyl]sulfanyl}methyl)-5-cyano-4-(3-pyridinyl)-4H-pyran-3-carboxylate | [s]1c(ccc1)c2nc(c(c(c2)c5ccc(cc5)Cl)C#N)SCC3=C(C(C(=C(O3)N)C#N)c4cnccc4)C(=O)OCC | C31H22O3N5Cl1S2 |
| 27348341 | Vitas-M Laboratory, Ltd. | STK528432 | N-(4-fluorobenzyl)-N'-(1-naphthyl)-N-[4-(9H-thioxanthen-9-yl)phenyl]urea | Fc1ccc(cc1)CN(c4ccc(cc4)C5c6c(cccc6)Sc7c5cccc7)C(=O)Nc2c3c(ccc2)cccc3 | C37H27O1N2S1F1 |
| 27558683 | Vitas-M Laboratory, Ltd. | STK532121 | 4-(3-methoxyphenyl)-1-(phenethylthio)-4H-spiro[benzo[h][1,2,4]triazolo[4,3-a]quinazoline-6,1'-cyclohexan]-5(7H)-one | S(CCc7ccccc7)c1[n]2c(nn1)N(C(=O)C4=C2c5c(cccc5)CC64CCCCC6)c3cc(ccc3)OC | C33H32O2N4S1 |
| 48658033 | Vitas-M Laboratory, Ltd. | STK597215 | 4-(3,4-dimethylphenyl)-1-(4-ethylphenyl)-N-(3-methylphenyl)-5,6,7,8-tetrahydro-2a,4a-diazacyclopenta[cd]azulene-2-carboxamide | [n]21c3c(c(c2C(=O)Nc6cc(ccc6)C)c5ccc(cc5)CC)CCCCN3C(=C1)c4cc(c(cc4)C)C | C34H35O1N3 |
| 28869109 | Vitas-M Laboratory, Ltd. | STK065789 | 4-[bis(5-hydroxy-3-methyl-1H-pyrazol-4-yl)methyl]-2-ethoxyphenyl 2-chlorobenzoate | Clc1c(cccc1)C(=O)Oc2c(cc(cc2)C(c4c([nH]nc4C)O)c3c([nH]nc3C)O)OCC | C24H23O5N4Cl1 |
| 27462543 | Vitas-M Laboratory, Ltd. | STK084760 | N-{2-methyl-1-[(2-naphthyloxy)acetyl]-1,2,3,4-tetrahydro-4-quinolinyl}-2-(2-naphthyloxy)-N-phenylacetamide | N3(C(CC(c7c3cccc7)N(c6ccccc6)C(=O)COc4cc5c(cc4)cccc5)C)C(=O)COc1cc2c(cc1)cccc2 | C40H34O4N2 |
| 30738164 | ChemDiv, Inc | 5867-4076 | N-[3-(1,3-benzothiazol-2-yl)phenyl]-3-[(5-phenyl[1,3]thiazolo[2,3-c][1,2,4]triazol-3-yl)sulfanyl]propanamide | S1c2[n](c(nn2)SCCC(=O)Nc4cc(ccc4)c5[s]c6c(n5)cccc6)C(=C1)c3ccccc3 | C26H19O1N5S3 |
| 40756209 | ChemDiv, Inc | C797-0522 | 1-[1-(2,5-dimethylbenzyl)-1H-benzimidazol-2-yl]-N-[2-(1H-indol-3-yl)ethyl]-4-piperidinecarboxamide | N4(CCC(CC4)C(=O)NCCc5c6c([nH]c5)cccc6)c1[n](c3c(n1)cccc3)Cc2c(ccc(c2)C)C | C32H35O1N5 |
| 40768166 | ChemDiv, Inc | C797-0834 | 4-({[3-(4-methoxybenzyl)-3H-imidazo[4,5-c]pyridin-2-yl]sulfanyl}methyl)-N-(4-methylbenzyl)benzamide | S(Cc4ccc(cc4)C(=O)NCc5ccc(cc5)C)c1[n](c3c(n1)ccnc3)Cc2ccc(cc2)OC | C30H28O2N4S1 |
| 61639904 | ChemDiv, Inc | C878-1784 | 3-[4-(2,3-dihydro-1H-indol-1-ylcarbonyl)benzyl]-2-[(4-methylbenzyl)sulfanyl]-3H-imidazo[4,5-c]pyridine | S(Cc6ccc(cc6)C)c1[n](c5c(n1)ccnc5)Cc2ccc(cc2)C(=O)N3CCc4c3cccc4 | C30H26O1N4S1 |
| 61602513 | ChemDiv, Inc | D016-1208 | 1-[5-{3-[4-(5-chloro-2-methylphenyl)-1-piperazinyl]-2-hydroxypropoxy}-2-methyl-1-(4-methylphenyl)-1H-indol-3-yl]ethanone | Clc1cc(c(cc1)C)N2CCN(CC2)CC(O)COc3cc4c([n](c(c4C(=O)C)C)c5ccc(cc5)C)cc3 | C32H36O3N3Cl1 |
| 30295813 | ChemDiv, Inc | C096-0092 | 2-(5-{[4-(2-methoxyphenyl)-1-piperazinyl]sulfonyl}-6-methyl-4-oxothieno[2,3-d]pyrimidin-3(4H)-yl)-N-methyl-N-[2-(2-pyridinyl)ethyl]acetamide | [S](=O)(=O)(N4CCN(CC4)c5c(cccc5)OC)c1c2c([s]c1C)N=CN(C2=O)CC(=O)N(CCc3ncccc3)C | C28H32O5N6S2 |
| 64190611 | ChemDiv, Inc | C794-1611 | 1-(5-{2-[(4-ethoxyanilino)methyl]-1H-pyrrol-1-yl}-1,3,4-thiadiazol-2-yl)-N-[2-(1H-indol-3-yl)ethyl]-4-piperidinecarboxamide | [s]1c(nnc1N4CCC(CC4)C(=O)NCCc5c6c([nH]c5)cccc6)[n]2c(ccc2)CNc3ccc(cc3)OCC | C31H35O2N7S1 |
| 111269525 | ChemDiv, Inc | F154-0105 | 4-({2-[(4-chlorobenzyl)sulfanyl]-3H-imidazo[4,5-c]pyridin-3-yl}methyl)-N-cyclooctylbenzamide | S(Cc5ccc(cc5)Cl)c1[n](c4c(n1)ccnc4)Cc2ccc(cc2)C(=O)NC3CCCCCCC3 | C29H31O1N4Cl1S1 |
| 111269527 | ChemDiv, Inc | F154-0109 | 4-({2-[(2-chloro-4-fluorobenzyl)sulfanyl]-3H-imidazo[4,5-c]pyridin-3-yl}methyl)-N-cyclooctylbenzamide | Fc1cc(c(cc1)CSc2[n](c5c(n2)ccnc5)Cc3ccc(cc3)C(=O)NC4CCCCCCC4)Cl | C29H30O1N4Cl1S1F1 |
| 111269549 | ChemDiv, Inc | F154-0183 | 4-chlorobenzyl 3-[4-(2,3-dihydro-1H-indol-1-ylcarbonyl)benzyl]-3H-imidazo[4,5-c]pyridin-2-yl sulfide | S(Cc6ccc(cc6)Cl)c1[n](c5c(n1)ccnc5)Cc2ccc(cc2)C(=O)N3CCc4c3cccc4 | C29H23O1N4Cl1S1 |
| 111269574 | ChemDiv, Inc | F154-0254 | 4-({2-[(3-chlorobenzyl)sulfanyl]-3H-imidazo[4,5-c]pyridin-3-yl}methyl)-N-(2,4-dimethylphenyl)benzamide | S(Cc5cc(ccc5)Cl)c1[n](c4c(n1)ccnc4)Cc2ccc(cc2)C(=O)Nc3c(cc(cc3)C)C | C29H25O1N4Cl1S1 |
| 111269732 | ChemDiv, Inc | F154-0645 | N-(2-methylbenzyl)-4-[(2-{[3-(trifluoromethyl)benzyl]sulfanyl}-3H-imidazo[4,5-c]pyridin-3-yl)methyl]benzamide | FC(F)(F)c1cc(ccc1)CSc2[n](c5c(n2)ccnc5)Cc3ccc(cc3)C(=O)NCc4c(cccc4)C | C30H25O1N4S1F3 |
| 182707700 | ChemDiv, Inc | G430-1653 | (1-(2-(3,4-dimethylphenyl)pyrazolo[1,5-a]pyrazin-4-yl)piperidin-3-yl)(4-phenylpiperazin-1-yl)methanone | [n]21nc(cc2C(=NC=C1)N4CC(CCC4)C(=O)N5CCN(CC5)c6ccccc6)c3cc(c(cc3)C)C | C30H34O1N6 |
| 30335059 | ChemDiv, Inc | K781-0391 | 3-(4-(tert-butyl)benzamido)-N-(3-chlorobenzyl)-4-(8-oxo-1,5,6,8-tetrahydro-2H-1,5-methanopyrido[1,2-a][1,5]diazocin-3(4H)-yl)benzamide | Clc1cc(ccc1)CNC(=O)c2cc(c(cc2)N4CC5CN6C(=CC=CC6=O)C(C4)C5)NC(=O)c3ccc(cc3)C(C)(C)C | C36H37O3N4Cl1 |
| 30335319 | ChemDiv, Inc | K781-1690 | ethyl 4-(4-(8-oxo-1,5,6,8-tetrahydro-2H-1,5-methanopyrido[1,2-a][1,5]diazocin-3(4H)-yl)-3-(3-(trifluoromethyl)benzamido)benzamido)piperidine-1-carboxylate | FC(F)(F)c1cc(ccc1)C(=O)Nc2c(ccc(c2)C(=O)NC6CCN(CC6)C(=O)OCC)N3CC4CN5C(=CC=CC5=O)C(C3)C4 | C34H36O5N5F3 |
| 30335447 | ChemDiv, Inc | K781-1931 | N-(5-(4-benzylpiperidine-1-carbonyl)-2-(8-oxo-1,5,6,8-tetrahydro-2H-1,5-methanopyrido[1,2-a][1,5]diazocin-3(4H)-yl)phenyl)-3-(trifluoromethyl)benzamide | FC(F)(F)c1cc(ccc1)C(=O)Nc2c(ccc(c2)C(=O)N6CCC(CC6)Cc7ccccc7)N3CC4CN5C(=CC=CC5=O)C(C3)C4 | C38H37O3N4F3 |
| 34713223 | **ChemDiv, Inc** | **K788-1241** | **N-(4-isopropylphenyl)-3-{[4-(2-methoxyphenyl)-1-piperazinyl]carbonyl}-4-oxo-1,4-dihydro-6-quinolinesulfonamide (G2iG)** | **[S](=O)(=O)(Nc5ccc(cc5)C(C)C)c1cc2c(ncc(c2O)C(=O)N3CCN(CC3)c4c(cccc4)OC)cc1** | **C30H32O5N4S1** |
| 111278730 | **ChemDiv, Inc** | **F228-0561** | **1-(5,6-dimethylfuro[2,3-d]pyrimidin-4-yl)-N-[2-(5-methyl-1H-indol-3-yl)ethyl]-3-piperidinecarboxamide (G2iK)** | **N3(CC(CCC3)C(=O)NCCc4c5c([nH]c4)ccc(c5)C)c1ncnc2[o]c(c(c21)C)C** | **C25H29O2N5** |
| 185953192 | ChemDiv, Inc | G435-0034 | (4-(2,5-dimethylphenyl)piperazin-1-yl)(1-(2-phenylpyrazolo[1,5-a]pyrazin-4-yl)piperidin-4-yl)methanone | [n]21nc(cc2C(=NC=C1)N4CCC(CC4)C(=O)N5CCN(CC5)c6c(ccc(c6)C)C)c3ccccc3 | C30H34O1N6 |
| 237221619 | ChemDiv, Inc | G435-0553 | (1-(2-(2,4-dimethylphenyl)pyrazolo[1,5-a]pyrazin-4-yl)piperidin-4-yl)(4-(2-methoxyphenyl)piperazin-1-yl)methanone | [n]21nc(cc2C(=NC=C1)N4CCC(CC4)C(=O)N5CCN(CC5)c6c(cccc6)OC)c3c(cc(cc3)C)C | C31H36O2N6 |
| 237187975 | ChemDiv, Inc | L390-0507 | N-(2-(1H-indol-3-yl)ethyl)-7-(3,4-dimethylphenyl)pyrazolo[1,5-a]pyrimidine-3-carboxamide | [n]21ncc(c2N=CC=C1c5cc(c(cc5)C)C)C(=O)NCCc3c4c([nH]c3)cccc4 | C25H23O1N5 |
| 237189315 | ChemDiv, Inc | L483-0680 | 4-(4-(4-benzylpiperidine-1-carbonyl)piperidin-1-yl)-6-ethoxyquinoline-3-carbonitrile | N3(CCC(CC3)C(=O)N4CCC(CC4)Cc5ccccc5)c1c2c(ncc1C#N)ccc(c2)OCC | C30H34O2N4 |
| 90394475 | ChemDiv, Inc | V005-9064 | 6-methoxy-2-[4-(trifluoromethoxy)benzoyl]-1-{5-[2-(trifluoromethyl)phenyl]-2-furyl}-2,3,4,9-tetrahydro-1H-beta-carboline | FC(F)(F)Oc1ccc(cc1)C(=O)N2CCc3c4c([nH]c3C2c5[o]c(cc5)c6c(cccc6)C(F)(F)F)ccc(c4)OC | C31H22O4N2F6 |
| 90317639 | ChemDiv, Inc | V007-0482 | 8-(4-{[(2-tert-butylphenyl)sulfanyl]methyl}benzoyl)-3-(2-isopropylphenoxy)-8-azabicyclo[3.2.1]octane | S(Cc2ccc(cc2)C(=O)N3C4CCC3CC(C4)Oc5c(cccc5)C(C)C)c1c(cccc1)C(C)(C)C | C34H41O2N1S1 |
| 121116441 | ChemDiv, Inc | V003-6571 | 4-fluoro-N-isobutyl-N-[2-oxo-2-({4-phenyl-1-[4-(trifluoromethyl)phenyl]-1H-imidazol-2-yl}amino)ethyl]benzamide | FC(F)(F)c1ccc(cc1)[n]2c(nc(c2)c4ccccc4)NC(=O)CN(CC(C)C)C(=O)c3ccc(cc3)F | C29H26O2N4F4 |
| 121285335 | ChemDiv, Inc | V007-7958 | 6-benzyl-4-{4-[(4-tert-butylphenyl)sulfonyl]-1-piperazinyl}-1-(3-chlorophenyl)-1H-pyrazolo[3,4-d]pyrimidine | [S](=O)(=O)(N2CCN(CC2)c3nc(nc5[n](ncc53)c6cc(ccc6)Cl)Cc4ccccc4)c1ccc(cc1)C(C)(C)C | C32H33O2N6Cl1S1 |
| 234347154 | ChemDiv, Inc | V008-7832 | N,3-bis(4-fluorobenzyl)-8-(trifluoromethyl)-2,3,4,4a,5,6-hexahydro-1H-pyrazino[1,2-a]quinoline-5-carboxamide | FC(F)(F)c1cc2c(cc1)N3C(CN(CC3)Cc5ccc(cc5)F)C(C2)C(=O)NCc4ccc(cc4)F | C28H26O1N3F5 |
| 49282656 | ChemDiv, Inc | V009-0402 | N-(4-tert-butylbenzyl)-2-[(4-chlorobenzyl)(cyclopropyl)amino]-N-[2-(1H-indol-3-yl)ethyl]acetamide | Clc1ccc(cc1)CN(C5CC5)CC(=O)N(CCc3c4c([nH]c3)cccc4)Cc2ccc(cc2)C(C)(C)C | C33H38O1N3Cl1 |
| 154638968 | ChemDiv, Inc | V009-3943 | 2-{[(4-tert-butylbenzyl)(3,3-diphenylpropyl)amino]methyl}-N-(2-furylmethyl)-1,3-thiazole-4-carboxamide | [s]1c(nc(c1)C(=O)NCc5[o]ccc5)CN(CCC(c4ccccc4)c3ccccc3)Cc2ccc(cc2)C(C)(C)C | C36H39O2N3S1 |
| 90386568 | ChemDiv, Inc | V011-3487 | 1-({1-[3',5'-bis(trifluoromethyl)[1,1'-biphenyl]-4-yl]-1H-imidazol-5-yl}carbonyl)-4-(4-tert-butylbenzyl)piperidine | FC(F)(F)c1cc(cc(c1)c2ccc(cc2)[n]3cncc3C(=O)N4CCC(CC4)Cc5ccc(cc5)C(C)(C)C)C(F)(F)F | C34H33O1N3F6 |
| 154930032 | ChemDiv, Inc | V014-0753 | 2-[[3-(benzyloxy)-2-hydroxypropyl](butyl)amino]-N-(4-tert-butylbenzyl)-N-[2-(1H-indol-3-yl)ethyl]acetamide | [nH]1c2c(c(c1)CCN(Cc4ccc(cc4)C(C)(C)C)C(=O)CN(CC(O)COCc3ccccc3)CCCC)cccc2 | C37H49O3N3 |
| 121204288 | ChemDiv, Inc | V014-1570 | 2-methyl-N-(2-{[1-(4-methylphenyl)-4-phenyl-1H-imidazol-2-yl]amino}-2-oxoethyl)-N-[2-(4-morpholinyl)ethyl]benzamide | [n]2(c(nc(c2)c5ccccc5)NC(=O)CN(CCN4CCOCC4)C(=O)c3c(cccc3)C)c1ccc(cc1)C | C32H35O3N5 |
| 234333487 | ChemDiv, Inc | V014-8901 | 1-benzhydryl-4-{[1-(2,5-dimethylphenyl)-3-(3-methoxyphenyl)-1H-pyrazol-5-yl]carbonyl}piperazine | [n]2(nc(cc2C(=O)N4CCN(CC4)C(c6ccccc6)c5ccccc5)c3cc(ccc3)OC)c1c(ccc(c1)C)C | C36H36O2N4 |
| 372736109 | ChemDiv, Inc | V015-4774 | (4-(2-fluorophenyl)piperazin-1-yl)(4-((5-isopropyl-4-p-tolyl-4H-1,2,4-triazol-3-ylthio)methyl)phenyl)methanone | Fc1c(cccc1)N2CCN(CC2)C(=O)c3ccc(cc3)CSc4[n](c(nn4)C(C)C)c5ccc(cc5)C | C30H32O1N5S1F1 |
| 154643154 | ChemDiv, Inc | V020-4178 | 4-fluoro-N-isobutyl-N-({1-[3-(trifluoromethyl)benzyl]-4-[3-(trifluoromethyl)phenyl]-3-pyrrolidinyl}methyl)benzamide | FC(F)(F)c1cc(ccc1)C2CN(CC2CN(CC(C)C)C(=O)c4ccc(cc4)F)Cc3cc(ccc3)C(F)(F)F | C31H31O1N2F7 |
| 344693638 | ChemDiv, Inc | V025-8637 | 3-({4-[(4-fluorophenyl)acetyl]-1-piperazinyl}methyl)-6-(3-methylphenyl)-2-phenylimidazo[1,2-a]pyridine | Fc1ccc(cc1)CC(=O)N2CCN(CC2)Cc3[n]4c(nc3c6ccccc6)C=CC(=C4)c5cc(ccc5)C | C33H31O1N4F1 |
| 121097081 | **ChemDiv, Inc** | **V025-9097** | **N-benzyl-N-(2-{[4-(4-chlorophenyl)-1-(3,4-dimethoxyphenyl)-1H-imidazol-2-yl]amino}-2-oxoethyl)-4-methylbenzamide (G2iB)** | **Clc1ccc(cc1)c2nc([n](c2)c5cc(c(cc5)OC)OC)NC(=O)CN(Cc4ccccc4)C(=O)c3ccc(cc3)C** | **C34H31O4N4Cl1** |
| 344695929 | ChemDiv, Inc | V026-6880 | 2-[(4-{[2-(4-chlorophenyl)-6-(3-methylphenyl)imidazo[1,2-a]pyridin-3-yl]methyl}-1-piperazinyl)sulfonyl]benzonitrile | [S](=O)(=O)(N2CCN(CC2)Cc3[n]4c(nc3c6ccc(cc6)Cl)C=CC(=C4)c5cc(ccc5)C)c1c(cccc1)C#N | C32H28O2N5Cl1S1 |
| 344693629 | ChemDiv, Inc | V027-0500 | 2-(4-methylphenyl)-3-({4-[4-(trifluoromethyl)benzoyl]-1-piperazinyl}methyl)-6-[3-(trifluoromethyl)phenyl]imidazo[1,2-a]pyridine | FC(F)(F)c1cc(ccc1)C2=C[n]3c(nc(c3CN5CCN(CC5)C(=O)c6ccc(cc6)C(F)(F)F)c4ccc(cc4)C)C=C2 | C34H28O1N4F6 |
| 344693828 | ChemDiv, Inc | V027-0530 | 6-(3-ethoxyphenyl)-3-{[4-(3-fluorobenzoyl)-1-piperazinyl]methyl}-2-(4-methylphenyl)imidazo[1,2-a]pyridine | Fc1cc(ccc1)C(=O)N2CCN(CC2)Cc3[n]4c(nc3c6ccc(cc6)C)C=CC(=C4)c5cc(ccc5)OCC | C34H33O2N4F1 |
| 344693645 | ChemDiv, Inc | V027-1389 | 3-({4-[(4-fluorophenyl)acetyl]-1-piperazinyl}methyl)-2-(3-methoxyphenyl)-6-[3-(trifluoromethyl)phenyl]imidazo[1,2-a]pyridine | FC(F)(F)c1cc(ccc1)C2=C[n]3c(nc(c3CN5CCN(CC5)C(=O)Cc6ccc(cc6)F)c4cc(ccc4)OC)C=C2 | C34H30O2N4F4 |
| 344693525 | ChemDiv, Inc | V027-1402 | 6-(1-benzothien-2-yl)-2-(4-chlorophenyl)-3-[(4-{[3-(trifluoromethyl)phenyl]sulfonyl}-1-piperazinyl)methyl]imidazo[1,2-a]pyridine | FC(F)(F)c1cc(ccc1)[S](=O)(=O)N2CCN(CC2)Cc3[n]4c(nc3c7ccc(cc7)Cl)C=CC(=C4)c5[s]c6c(c5)cccc6 | C33H26O2N4Cl1S2F3 |
| 154821145 | ChemDiv, Inc | V027-1461 | 2-(4-chlorophenyl)-3-({4-[(4-fluorophenyl)sulfonyl]-1-piperazinyl}methyl)-6-phenylimidazo[1,2-a]pyridine | Fc1ccc(cc1)[S](=O)(=O)N2CCN(CC2)Cc3[n]4c(nc3c6ccc(cc6)Cl)C=CC(=C4)c5ccccc5 | C30H26O2N4Cl1S1F1 |
| 344693835 | ChemDiv, Inc | V027-4749 | 4-(6-(1-benzothien-2-yl)-3-{[4-(3-fluorobenzoyl)-1-piperazinyl]methyl}imidazo[1,2-a]pyridin-2-yl)phenyl methyl ether | Fc1cc(ccc1)C(=O)N2CCN(CC2)Cc3[n]4c(nc3c7ccc(cc7)OC)C=CC(=C4)c5[s]c6c(c5)cccc6 | C34H29O2N4S1F1 |
| 90368846 | ChemDiv, Inc | V027-7169 | 1-(diphenylacetyl)-4-(3,5-diphenyl-1H-pyrazol-1-yl)piperidine | [n]4(nc(cc4c6ccccc6)c5ccccc5)C1CCN(CC1)C(=O)C(c3ccccc3)c2ccccc2 | C34H31O1N3 |
| 344696025 | ChemDiv, Inc | V029-3339 | 6-(3-ethoxyphenyl)-2-(4-fluorophenyl)-3-({4-[(4-fluorophenyl)sulfonyl]-1-piperazinyl}methyl)imidazo[1,2-a]pyridine | Fc1ccc(cc1)[S](=O)(=O)N2CCN(CC2)Cc3[n]4c(nc3c6ccc(cc6)F)C=CC(=C4)c5cc(ccc5)OCC | C32H30O3N4S1F2 |
| 332647939 | ChemDiv, Inc | V029-3540 | 3-[4-(benzyloxy)phenyl]-3-[1-(4-fluorobenzyl)-1H-indol-3-yl]-N-[2-(2-pyridinyl)ethyl]propanamide | Fc1ccc(cc1)C[n]2c3c(c(c2)C(CC(=O)NCCc6ncccc6)c4ccc(cc4)OCc5ccccc5)cccc3 | C38H34O2N3F1 |
| 344693512 | ChemDiv, Inc | V029-8627 | 3-{[4-(4-tert-butylbenzoyl)-1-piperazinyl]methyl}-6-(2,5-dimethylphenyl)-2-phenylimidazo[1,2-a]pyridine | [n]21c(nc(c2CN5CCN(CC5)C(=O)c6ccc(cc6)C(C)(C)C)c4ccccc4)C=CC(=C1)c3c(ccc(c3)C)C | C37H40O1N4 |
| 154819933 | ChemDiv, Inc | V029-9895 | 8-[(4-{[2-(4-chlorophenyl)-6-phenylimidazo[1,2-a]pyridin-3-yl]methyl}-1-piperazinyl)sulfonyl]quinoline | [S](=O)(=O)(N3CCN(CC3)Cc4[n]5c(nc4c7ccc(cc7)Cl)C=CC(=C5)c6ccccc6)c1c2ncccc2ccc1 | C33H28O2N5Cl1S1 |
| 332651912 | **ChemDiv, Inc** | **V030-0484** | **3-(5-chloro-1H-indol-3-yl)-3-[3-(4-chlorophenoxy)phenyl]-N-[2-(4-morpholinyl)ethyl]propenamide (G2iI)** | **Clc1ccc(cc1)Oc2cc(ccc2)C(CC(=O)NCCN5CCOCC5)c3c4c([nH]c3)ccc(c4)Cl** | **C29H29O3N3Cl2** |
| 372880764 | ChemDiv, Inc | V030-1419 | (3-((4-(4-fluorophenyl)-5-(furan-2-yl)-4H-1,2,4-triazol-3-ylthio)methyl)phenyl)(4-(2-fluorophenyl)piperazin-1-yl)methanone | Fc1c(cccc1)N2CCN(CC2)C(=O)c3cc(ccc3)CSc4[n](c(nn4)c6[o]ccc6)c5ccc(cc5)F | C30H25O2N5S1F2 |
| 344693633 | ChemDiv, Inc | V030-2174 | 6-(1-benzothien-2-yl)-2-(4-chlorophenyl)-3-({4-[4-(trifluoromethyl)benzoyl]-1-piperazinyl}methyl)imidazo[1,2-a]pyridine | FC(F)(F)c1ccc(cc1)C(=O)N2CCN(CC2)Cc3[n]4c(nc3c7ccc(cc7)Cl)C=CC(=C4)c5[s]c6c(c5)cccc6 | C34H26O1N4Cl1S1F3 |
| 344696239 | ChemDiv, Inc | V030-2573 | 1-{4-[3-({4-[(2-chloro-5-nitrophenyl)sulfonyl]-1-piperazinyl}methyl)-2-phenylimidazo[1,2-a]pyridin-6-yl]phenyl}ethanone | [S](=O)(=O)(N2CCN(CC2)Cc3[n]4c(nc3c6ccccc6)C=CC(=C4)c5ccc(cc5)C(=O)C)c1c(ccc(c1)[N+](=O)[O-])Cl | C32H28O5N5Cl1S1 |
| 344696238 | ChemDiv, Inc | V030-2628 | 3-({4-[(2-chloro-5-nitrophenyl)sulfonyl]-1-piperazinyl}methyl)-2-(3-methoxyphenyl)-6-(3-methylphenyl)imidazo[1,2-a]pyridine | [S](=O)(=O)(N2CCN(CC2)Cc3[n]4c(nc3c6cc(ccc6)OC)C=CC(=C4)c5cc(ccc5)C)c1c(ccc(c1)[N+](=O)[O-])Cl | C32H30O5N5Cl1S1 |
| 344696332 | ChemDiv, Inc | V030-2674 | 2-(4-fluorophenyl)-3-({4-[(3-nitrophenyl)sulfonyl]-1-piperazinyl}methyl)-6-[3-(trifluoromethyl)phenyl]imidazo[1,2-a]pyridine | FC(F)(F)c1cc(ccc1)C2=C[n]3c(nc(c3CN5CCN(CC5)[S](=O)(=O)c6cc(ccc6)[N+](=O)[O-])c4ccc(cc4)F)C=C2 | C31H25O4N5S1F4 |
| 344696242 | ChemDiv, Inc | V030-2685 | 3-({4-[(2-chloro-5-nitrophenyl)sulfonyl]-1-piperazinyl}methyl)-2-(4-fluorophenyl)-6-(3-methylphenyl)imidazo[1,2-a]pyridine | Fc1ccc(cc1)c2nc3[n](c2CN5CCN(CC5)[S](=O)(=O)c6c(ccc(c6)[N+](=O)[O-])Cl)C=C(C=C3)c4cc(ccc4)C | C31H27O4N5Cl1S1F1 |
| 332657079 | ChemDiv, Inc | V030-2767 | 3-[3-(4-chlorophenoxy)phenyl]-3-(1H-indol-3-yl)-N-(4-pyridinylmethyl)propanamide | Clc1ccc(cc1)Oc2cc(ccc2)C(CC(=O)NCc5ccncc5)c3c4c([nH]c3)cccc4 | C29H24O2N3Cl1 |
| 344696288 | ChemDiv, Inc | V030-5887 | N-{4-[(4-{[6-(3-chlorophenyl)-2-(4-chlorophenyl)imidazo[1,2-a]pyridin-3-yl]methyl}-1-piperazinyl)sulfonyl]phenyl}acetamide | [S](=O)(=O)(N2CCN(CC2)Cc3[n]4c(nc3c6ccc(cc6)Cl)C=CC(=C4)c5cc(ccc5)Cl)c1ccc(cc1)NC(=O)C | C32H29O3N5Cl2S1 |
| 344693611 | ChemDiv, Inc | V030-8097 | 3-({4-[(benzyloxy)acetyl]-1-piperazinyl}methyl)-6-(2,5-dimethylphenyl)-2-(4-nitrophenyl)imidazo[1,2-a]pyridine | [N+](=O)([O-])c1ccc(cc1)c2nc3[n](c2CN5CCN(CC5)C(=O)COCc6ccccc6)C=C(C=C3)c4c(ccc(c4)C)C | C35H35O4N5 |
| 117452839 | ChemDiv, Inc | L227-0253 | 1-(2-(4-chlorophenyl)-5-methylene-4,5-dihydropyrazolo[1,5-a]pyrimidin-7-yl)-N-((3-methyl-1H-pyrazol-4-yl)methyl)piperidine-4-carboxamide | Clc1ccc(cc1)c2n[n]3c(c2)NC(=C)C=C3N4CCC(CC4)C(=O)NCc5c[nH]nc5C | C24H26O1N7Cl1 |
| 40717273 | ChemDiv, Inc | C380-0602 | 10-(2-chloro-6-fluorobenzyl)-8-(3,4-dihydro-2(1H)-isoquinolinylcarbonyl)dibenzo[b,f][1,4]thiazepin-11(10H)-one | Fc1c(c(ccc1)Cl)CN2c3c(ccc(c3)C(=O)N5CCc6c(cccc6)C5)Sc4c(cccc4)C2=O | C30H22O2N2Cl1S1F1 |
